# Calpain-mediated tau fragmentation is altered in Alzheimer’s disease progression

**DOI:** 10.1038/s41598-018-35130-y

**Published:** 2018-11-13

**Authors:** Hsu-Hsin Chen, Peter Liu, Paul Auger, Seung-Hye Lee, Oskar Adolfsson, Lorianne Rey-Bellet, Julien Lafrance-Vanasse, Brad A. Friedman, Maria Pihlgren, Andreas Muhs, Andrea Pfeifer, James Ernst, Gai Ayalon, Kristin R. Wildsmith, Thomas G. Beach, Marcel P. van der Brug

**Affiliations:** 1 Biomarker Discovery, Genentech, Inc., 1 DNA Way, South San Francisco, CA USA; 2 Microchemistry, Proteomics and Lipidomics, Genentech, Inc., 1 DNA Way, South San Francisco, CA USA; 3 Biomarker Development, Genentech, Inc., 1 DNA Way, South San Francisco, CA USA; 4 Neuroscience, Genentech, Inc., 1 DNA Way, South San Francisco, CA USA; 5 AC Immune SA, EPFL Innovation Park, Building B, CH-1015 Lausanne, Switzerland; 6 Protein Chemistry, Genentech, Inc., 1 DNA Way, South San Francisco, CA USA; 7 Banner Sun Health Research Institute, 10515W Santa Fe Drive, Sun City, AZ USA; 8 Present Address: Therapeutics Division, Clover Health, 22 4th Street, San Francisco, CA USA

## Abstract

The aggregation of intracellular tau protein is a major hallmark of Alzheimer’s disease (AD). The extent and the stereotypical spread of tau pathology in the AD brain are correlated with cognitive decline during disease progression. Here we present an in-depth analysis of endogenous tau fragmentation in a well-characterized cohort of AD and age-matched control subjects. Using protein mass spectrometry and Edman degradation to interrogate endogenous tau fragments in the human brain, we identified two novel proteolytic sites, G323 and G326, as major tau cleavage events in both normal and AD cortex. These sites are located within the sequence recently identified as the structural core of tau protofilaments, suggesting an inhibitory mechanism of fibril formation. In contrast, a different set of novel cleavages showed a distinct increase in late stage AD. These disease-associated sites are located outside of the protofilament core sequence. We demonstrate that calpain 1 specifically cleaves at both the normal and diseased sites *in vitro*, and the site selection is conformation-dependent. Monomeric tau is predominantly cleaved at G323/G326 (normal sites), whereas oligomerization increases cleavages at the late-AD-associated sites. The fragmentation patterns specific to disease and healthy states suggest novel regulatory mechanisms of tau aggregation in the human brain.

## Introduction

Neurofibrillary tangles (NFTs) and senile plaques are the histopathological hallmarks of Alzheimer’s disease (AD). NFTs are intracellular protein aggregates of Microtubule Associated Protein Tau (MAPT), whereas senile plaques form extracellularly from Amyloid β peptides. While each of these features is associated with AD progression, post-mortem histological data and PET imaging studies showed a high degree of correlation between cognitive decline and NFT as opposed to amyloid burden^1–4^ and the co-localization of tau pathology with neuronal degeneration. Tau pathology is also the main feature of a number of other neurodegenerative diseases, collectively referred to as tauopathies. NFTs are observed in a subtype of frontotemporal dementia (FTDP-17)^5^ and chronic traumatic encephalopathy (CTE)^6^. Other tauopathies include Pick’s disease, progressive supranuclear palsy (PSP), corticobasal dementia (CBD) and argyrophilic grain disease, in which heterogeneous types of tau aggregates are present in respective brain areas undergoing degeneration. The molecular and structural changes of tau in these diseases are considered the main driver of functional circuitry loss and neuronal degeneration. While no causal mutations in the *MAPT* locus have been found for sporadic or familial AD, 30+ missense mutations have been confirmed as pathogenic in FTDP-17^7^. Transgenic mice harboring these MAPT mutations are widely employed as *in vivo* models for tauopathies.

**Figure 1:**
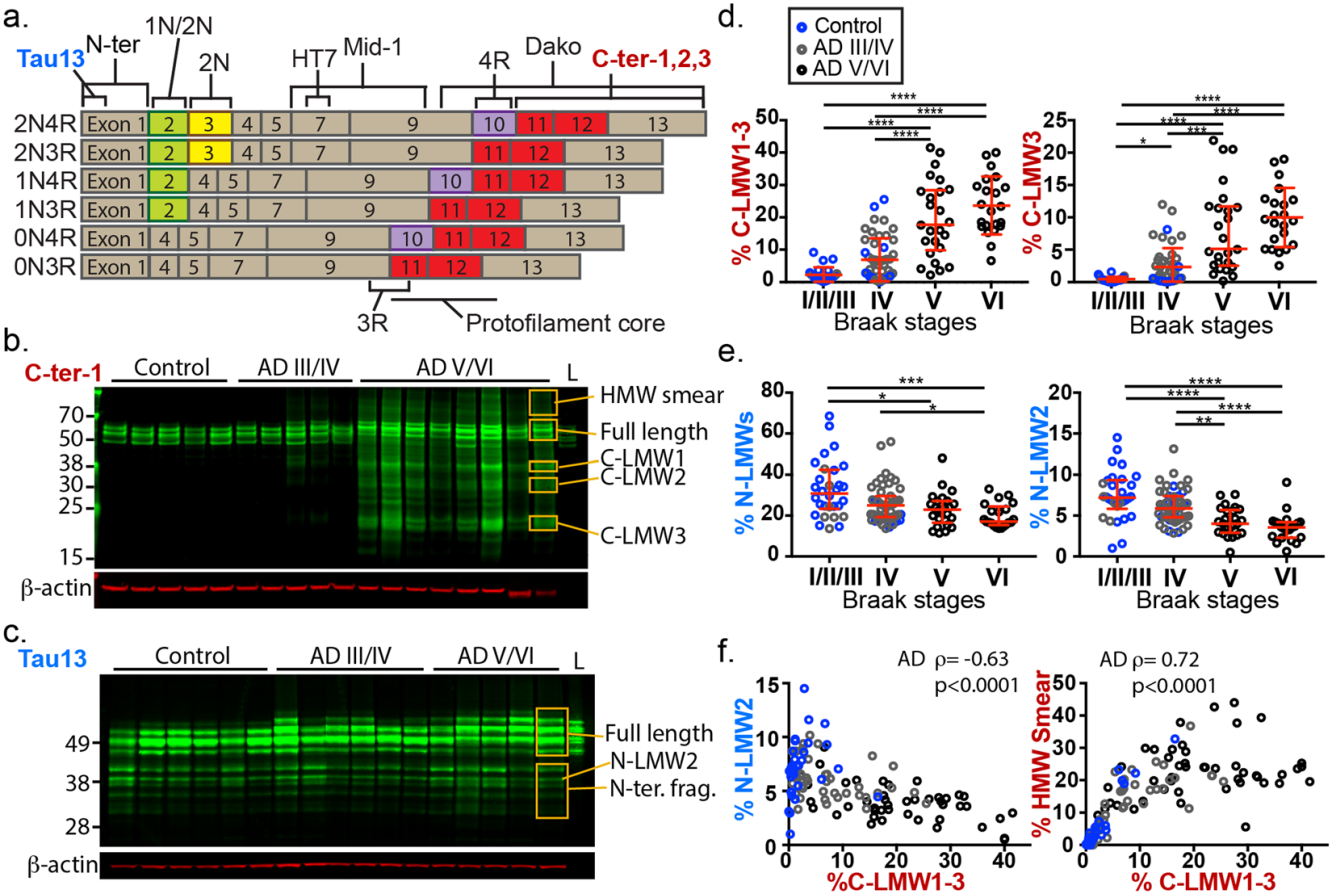
Shifts in tau fragment composition in cortical tissue during Alzheimer’s disease progression. (**a**) Schematics of epitope location and isoform specificity of tau antibodies used in this study. (**b**) Representative immunoblot for quantification of C-terminal tau protein fragments in AD and control brain lysates from human fusiform gyrus. 3 groups of major fragment bands (MW = 37 kDa, 30 kDa and 23 kDa, orange boxes) as well as full length tau and high molecular weight smear were quantified. (**c**) Representative immunoblot for quantification of N-terminal tau protein fragments in AD and control patient brain lysates of the fusiform gyrus. Individual full-length (four) and fragment bands (six) within orange-boxed areas are quantified. (**d**) Percentage of C-terminal tau fragments across Braak stages. Patients were re-classified based on Braak stages (histopathological designation) instead of clinical diagnosis (control vs. AD). Data point colors reflect original patient grouping. Left, sum %C-LMW1, 2 and 3; right, %C-LMW3 (lowest p value among C-terminal fragments). Braak I/II/III, N = 26 (20 Control, 6 AD); Braak IV, N = 49(12 Control, 37 AD), Braak V, N = 25 (all AD); Braak VI, N = 21 (all AD). (**e**) Percentage of N- terminal tau fragments across Braak stages. Left, sum % of all N-LMWs; right, N-LMW2 (lowest p value among N-terminal fragments). Braak I/II/III, N = 30 (23 Control, 7 AD); Braak IV, N = 49 (12 Control, 39 AD), Braak V, N = 25 (all AD); Braak VI, N = 21 (all AD). Dunn’s multiple comparison test, *p < 0.05, **p < 0.01, ***p < 0.001, ****p < 0.0001. (**f**) Percentage of C-terminal fragments positively correlated with the percentage of high molecular weight smear and negatively correlated with %N-LMW2 (Spearman’s rank correlation). Bars in (**d**,**e**) represent median with interquartile range. Full length pictures of immunoblots in (**b,c)** are shown in Supplementary Fig. 4. All additional immunoblots used for quantification are shown in Supplementary Figs 5 and 6.

The *MAPT* locus encodes six isoforms of tau via alternative splicing of exons 2 and 3 in the N-terminal region and exon 10 in the C-terminal microtubule-binding domain (Fig. 1a). Each isoform contains 0–2 N-terminal motifs and 3 or 4 microtubule binding repeats (MTBR); all six isoforms are found in NFT. In the adult neuron, tau is predominantly localized to the axon whereas fibrils accumulate in the somatodendritic compartment. Tau in dispersed filament fraction from AD brain is hyper-phosphorylated^8^ and its phosphorylation reduces microtubule binding affinity and promotes mis-localization to the soma and dendrites (reviewed in^9^). Phospho-tau mis-localization precedes tau tangle formation in mouse models (reviewed in^10^), and is characteristic of pre-tangle stage pathology.

In AD brain, tau pathology develops in a stereotypic fashion and its severity is correlated with cognitive decline and disease progression. Based on the extent of NFT spread, post-mortem brains are classified into six Braak stages, ranging from sparse NFTs confined to the entorhinal cortex as Braak I to pervasive involvement of most neocortical areas as Braak VI^11^. In Braak 0 brains (no NFT), pre-tangle stages based on phospho-tau staining were defined in post-mortem brains and these are on average decades younger than brains with NFT^12,13^. The molecular changes leading up to NFT formation thus appear to take years to develop, and hints at potential endogenous mechanisms that counteract tau aggregation.

Growing evidence points toward additional post-translational modifications of tau affecting fibril formation and neurotoxicity. Of increasing interest is proteolytic cleavage, as a significant portion of endogenous tau exists in fragments <45 kDa in human brain tissue^14–17^. The exact identities of these fragments and their function, if any, are largely unknown. Proteolytic processing of tau would not only regulate the steady-state level of full-length protein, but also produce truncated forms that are potentially important for normal neuronal function and pathological mechanisms in neurodegeneration. A number of different tau fragments generated by various proteases have been reported in human disease and in murine models. Caspase-mediated cleavages of tau have been identified in human NFT by immunohistochemistry^18,19^. Truncations at D13 and D421 by caspases were detected in AD brains and the levels correlate with disease progression^20–22^. Cleavage at D314 by caspase 2 was shown to promote tau mis-sorting into dendritic spines and to exacerbate cognitive deficits in tau^P301L^ mice^15^. In tau^P301S^ mice, deficiency of lysosomal asparagine endopeptidase inhibited the formation of tau1–368 fragment and alleviated tauopathy and synaptic defects^23^. Calpains were found to be abnormally activated in AD brains^24–26^, and in cultured hippocampal neurons, generate a toxic 17kD tau fragment in response to Aβ aggregate treatment^27,28^. Several other proteases, including HtrA1^29^ and cathepsins^30^, were also implicated in tau fragmentation with unclear functional relevance.

Tau fragments were also found to serve neuroprotective functions in mouse and *in vitro* models. Overexpression of the N-terminal portion of tau (1–225) was shown to alleviate Aβ-induced memory deficit and early mortality in transgenic APP mice^31^. The proposed mechanism is that tau^1–225^ sequesters Fyn kinase and prevents its entry into postsynaptic dendritic spines, destabilizes PSD-95 complexes and prevents excitatory neurotoxicity. Recent studies also indicate that the N-terminal domain of tau interacts with and affects the mobility of presynaptic vesicles, and expression of an N-terminal tau fragment can rescue the presynaptic effects of tau^P301L^ ^32^. While these artificial fragments are different from those found in the human brain, the findings suggest a potential role of proteolytic tau processing in counteracting neurodegeneration.

The propagation of tau pathology through the neural network is thought to follow a prion-like model (reviewed in^33^), in which “seeds” of pathological tau are transmitted from a diseased neuron to an unaffected one anterogradely, crossing the synapse either as secreted proteins or enclosed in exosomes^34^. Once taken up by the postsynaptic neuron, the seeds presumably recruit normal tau, induce conformational changes that promote aggregation, and in turn generate more seeds to infect the next neuron. Indeed, *in vitro* and *in vivo* tau seeds can propagate in cell-based systems and initiate tau pathology spread in transgenic mouse brain. Furthermore, tau fibrils purified from patient tissue of different tauopathies retain strain-specific phenotypes through successive propagation both *in vitro*^35,36^ and *in vivo*^37,38^. The prion-like spread model is the basis of tau immunotherapy approaches, which have been demonstrated to reduce tau pathology in tau^P301L^ mice^39^. While oligomers of tau with as few as 3 units can serve as seeding entities *in vitro*^40^, it is yet unclear which tau species constitute the endogenous seeds in the interstitial space. Tau is highly fragmented in the CSF from both control and AD patients^41^, and a recent study demonstrated with stable isotope labeling that tau protein is truncated in both human CNS and iPSC-derived neurons, and actively released from the latter^42^, lending to the possibility for truncated tau as candidates for the endogenous seeds.

In this study we sought to fill the need for a comprehensive catalog of tau fragments in the human brain. We analyzed the disease-associated changes in the tau fragment composition during AD progression and defined two distinct sets of novel calpain cleavage sites reflecting normal vs. diseased states. We showed that *in vitro*, the selection of disease-associated cleavage sites is dependent on the oligomerization of tau. Our results reveal a differential tau processing mechanism between the normal and aggregated tau with implications in tau protein quality control and pathology propagation.

## Results

### Shift in tau fragmentation patterns in late stage Alzheimer’s cortex

To analyze tau fragmentation in AD, we performed immunoblotting with C-terminal specific (in-house C-ter-1) and N-terminal specific (Tau13) tau antibodies on post-mortem fusiform gyrus lysate from 35 control (32 for C-terminal analysis), 46 AD Braak III/IV (43 for C-terminal analysis) and 46 AD Braak V/VI patients (see Methods and Table 1). The epitope locations of antibodies used in this work are shown in Fig. 1a. We found a group of C-terminal tau fragments migrating at 20–40 kDa predominantly in late Braak stage patients (Fig. 1b). In contrast, the N-terminal tau fragments (6 bands, 28–43 kDa) are detected at high levels across all patients (control, 27%, AD III/IV, 25% and AD V/VI, 20%, median values), with no obvious differences in pattern or apparent molecular weights across patient groups (Fig. 1c).

**Table 1:** Demographic of the patient cohort.

	Control (n = 35)	AD III/IV (n = 46)	AD V/VI (n = 46)
Gender (male/female)	21/13	20/26	29/17
Expired age	84.4 ± 6.4	89.6 ± 5.8	82.8 ± 9.1
ApoE4 (0/1/2)	27/8/0	26/18/1^a^	22/22/2
Disease duration (yr)	N/A	6.7 ± 4.0	7.6 ± 3.8
Last MMSE	28.5 ± 1.3	19.0 ± 7.4	10.5 ± 7.8
PMI (hr)	2.75 ± 0.68	2.79 ± 0.80	2.87 ± 0.64

^a^ApoE genotype is unknown for one patient.

We quantified the 3 major C-terminal fragments enriched in late stage AD patients, C-LMW1, 2 and 3 (37, 30 and 23 kDa) and normalized against total C-terminal tau signal (see Methods). The proportions of C-terminal fragments were significantly higher in AD V/VI samples versus the control or AD III/IV samples (p < 0.0001, Supplementary Fig. 1a). The percentages of total N-terminal tau fragments, on the contrary, showed a slight but significant decrease in late stage (V/VI) AD patients compared to control (p = 0.001) or early stage (III/IV) AD patients (p = 0.015). The stark difference between N- and C-terminal fragments suggests two different types of tau cleavage: a normal proteolytic mechanism shared by all subjects, control and AD alike, and a disease-associated one mainly in late-stage AD.

Patient grouping is based on both clinical diagnosis and histopathology, and the control subjects exhibit a wide range of tau pathology (Braak I-IV, Table 2). To specifically examine the correlation between fragmentation and tau pathology, we reanalyzed the extent of fragmentation solely against tau pathology. With patients grouped by Braak stages regardless of diagnosis (Table 2), we again observed a clear increase in C-LMW1, 2 and 3 as well as a significant decrease in N-terminal fragments with increasing severity of tau pathology (Fig. 1d,e). Similarly, %C-LMW1-3 showed positive rank correlation with total tangle score from 5 cortical areas (Supplementary Fig. 1b), whereas %N-LMWs (1–6) showed the opposite trend (ρ = −0.29, p = 0.005). Of the individual N-terminal fragments, the differences between patient groups/Braak stages as well as the negative correlation with total tangle score are most significant in band N-LMW2 (Supplementary Fig. 1b).

**Table 2:** Braak stages of the patient cohort.

Braak stages	Control (n = 35)	AD III/IV (n = 46)	AD V/VI (n = 46)
I	2	0	0
II	4	0	0
III	17	7	0
IV	12	39	0
V	0	0	25
VI	0	0	21

Importantly, %C-LMW1-3 and %N-LMW2 are negatively correlated to each other (Fig. 1f, left) and %C-LMW1-3, but not %N-LMW2, showed strong correlation with the amount of signal in the high molecular weight (HMW) tau signal smear, which likely represents partially denatured tau aggregates (Fig. 1f, right). None of the N- or C- terminal fragments showed correlation with post-mortem interval (data not shown), indicating the changes in fragmentation pattern is unlikely an overall degradation artifact.

Consistent with the tau pathology correlations, %C-LMW1-3 and %N-LMW2 also correlated in rank with the last Mini-Mental State Examination (MMSE) score and the age of dementia diagnosis (Supplementary Fig. 1c). The fragments, however, showed only weak correlation with amyloid plaque scores and no correlation with other clinical and histopathological measures including cerebral amyloid angiopathy scores, infarct volume (cortical and subcortical), motor UPDRS scores or disease duration (data not shown). Overall, high C-LMW1-3 and low N-LMW2 are associated with higher tau pathology and lower cognitive function.

### Characterization of tau fragments in the normal cortex

**Figure 2:**
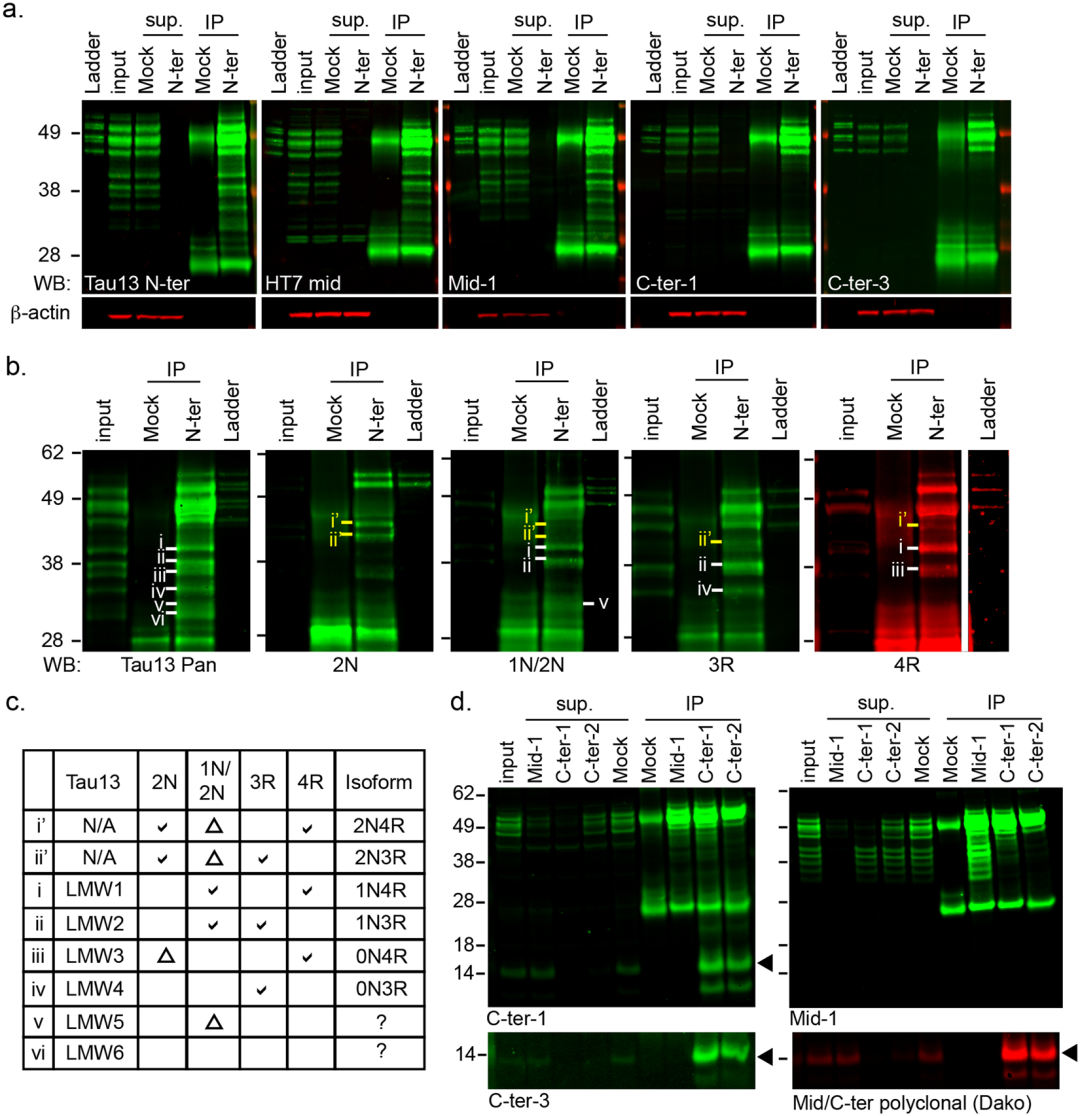
Characterization of normal tau fragmentation. (**a**) Immunoprecipitation of N-terminal-intact tau followed by immunoblotting with region-specific tau antibodies in human fusiform gyrus cortex lysate (AD Braak III/IV). Ladder, full length recombinant tau (6 isoforms); input, 1/10 of lysate for IP; Mock, mock IP with normal IgG; N-ter, tau IP with in-house N-terminal specific antibody N-ter; sup, IP supernatant; IP, eluent of IP from 100 μg brain lysate. (**b**) Isoform-specific immunoblotting of N-terminal-intact tau in human control frontal cortex lysate. Lanes: input, 1/10 of lysate for IP; Mock, mock IP with normal IgG, N-ter, tau IP with N-ter antibody, Ladder, full length recombinant tau (6 isoforms). The ladder band in 4 R is on the same gel but from a longer exposure due to low signal. (**c**) Table summary of N-terminal fragment isoform identification from (**b**) Bands i-vi are detected with Tau13 immunoblotting, and bands i’ and ii’ are new fragments detected with 2 N antibodies (in house). (**d**) Immunoprecipitation followed by immunoblotting of tau protein with C-terminal domain from human control frontal cortical lysate. Input, 1/10 of brain lysate for IP; Mock, mock IP with normal mouse IgG; Mid-1, tau IP with in house mid-domain specific antibody Mid-1; C-ter-1 and C-ter-2, tau IP with in-house C-terminal-specific antibodies C-ter-1 and C-ter-2; sup, IP supernatant; IP; eluent of IP from 100 μg brain lysate. Black arrowheads, 14 kDa C-LMW4 bands. Full length figures of all cropped blots are shown in Supplementary Fig. 7.

Our immunoblotting data indicate a normal tau cleavage event that occurs in both control and AD patients, as well as cleavages that are enriched in late Braak stages. To identify the normal cleavage site(s), we began by characterizing the N-terminal fragments that were detected across all samples. Full-length and N-terminal fragments of tau in cortical lysates were immunoprecipitated with monoclonal antibodies against the N-terminus (in-house N-ter) and immunoblotted with epitope-mapped monoclonal tau antibodies. Mid-domain specific antibodies (HT7 and Mid-1) detected all six major N-terminal fragments, whereas C-terminal specific antibodies did not (Fig. 2a). Immunodepletion of tau in brain lysates with N-ter also depleted mid-domain signal and *vice versa* (data not shown), indicating no prevalent cleavage events between the N-terminus and mid-domain. C-terminal specific tau antibodies immunoprecipitate full length tau isoforms but not the 28-45 kDa N-terminal fragments. Similar results were obtained from pooled and single-sample fusiform gyrus lysates of both control and AD patients, as well as frontal cortex lysates from control patients (data not shown).

To further characterize the individual N-terminal fragments, especially N-LMW2, we performed isoform-specific immunoblotting on immunoprecipitated tau fragments. Monoclonal antibodies specific to exon 3 (2N-specific) and exon 2 (1 N/2N-specific) were generated and epitope mapped in house (data not shown), and along with commercially available 3R and 4R antibodies were confirmed for isoform specificity using recombinant tau ladder (Fig. 2b, Ladder lane). Tau fragments immunoprecipitated from control frontal cortical lysate were interrogated with the isoform-specific antibodies (Fig. 2b). Isoform-specific signals of most tau bands in the lysate are insufficient and require IP enrichment for detection. With this data we were able to assign isoform origin for 4 out of the 6 major N-LMWs from the Tau13 immunoblot (bands i-iv, 0 N and 1 N isoforms, Fig. 2c). N-LMW2 is annotated as a derivative of 1N3R tau, which is expressed at higher levels and its abundance likely contributes to a higher signal-to-noise ratio than other fragments, and thus higher correlation to clinical and pathological parameters. Aside from the 6 N-LMWs, two additional tau fragments were detected with the 2N-specific antibodies above N-LMW1 in the immunoprecipitation (bands i’ and ii’): these are the major 2 N fragment bands detected, present at lower levels as are the full-length isoforms of origin. Together these results show that the normal mode of tau cleavage occurs in all 6 isoforms, and the cleavage sites, likely shared, are located C-terminal to residues encoded in exon 10, the last alternatively spliced exon.

**Figure 3:**
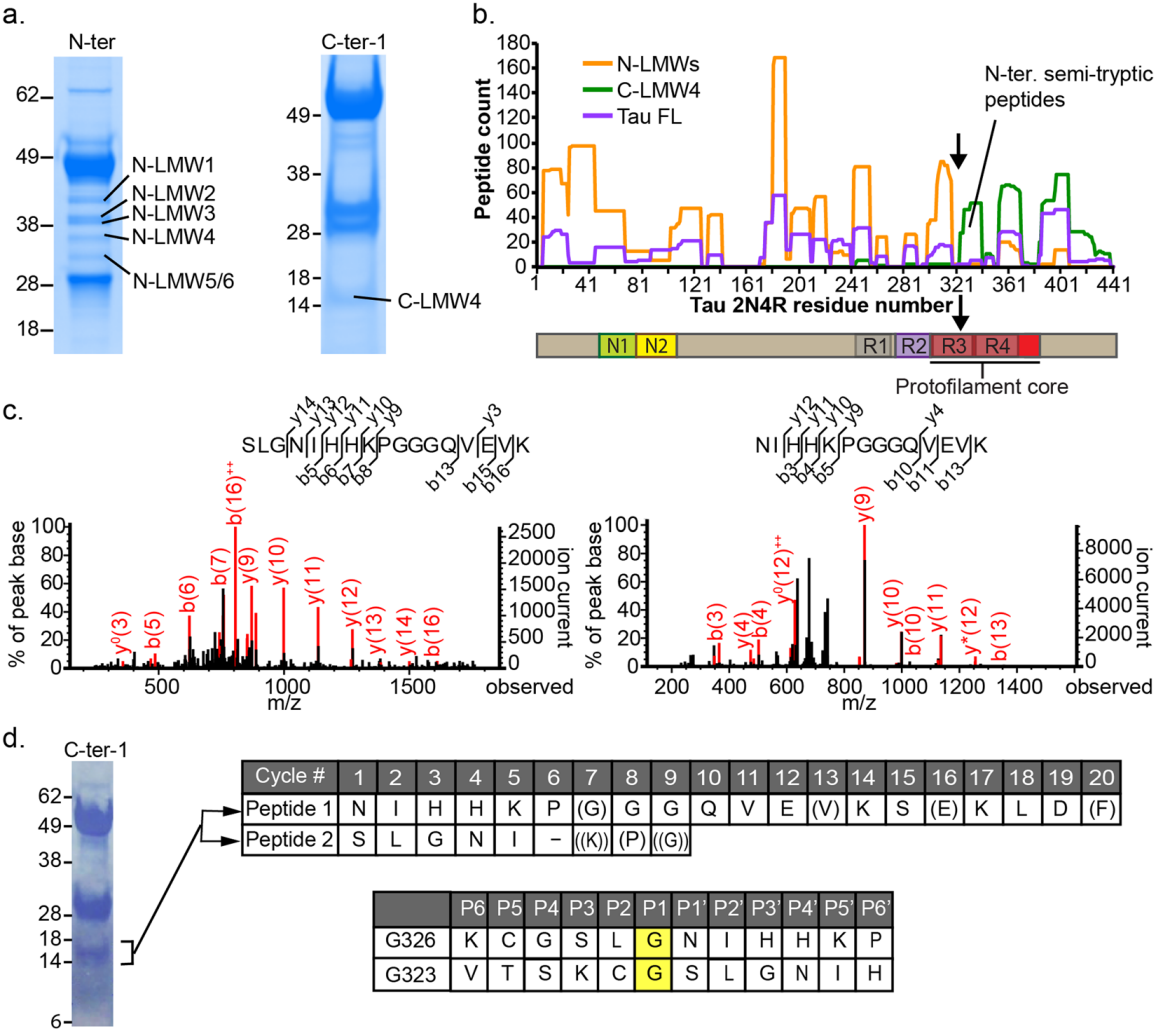
Identification of tau cleavage sites in normal cortex. (**a**) Isolation of tau fragment bands for tandem protein mass spectrometry identification of N- and C-terminal tau fragments from control human cortex. SafeBlue staining of immunoprecipitated tau using in house antibodies N-ter and C-ter-1. (**b**) Tryptic and semitryptic peptide counts from protein mass spectrometry experiment were mapped on 2N4R tau protein sequence. Black arrow, predicted cleavage site. (**c**). Protein mass spectrometry traces of the two major semitryptic peptides identified from C-LMW4 14kD fragment. (**d**) Identification of cleavage site by Edman degradation sequencing of the C-LMW4 immunoprecipitated from control frontal cortex. Left, Coomassie blue staining of C-ter-1 IP from control frontal cortex lysate; upper right, Two N-termini identified from Edman sequencing of the 14 kDa band, residues in brackets are of lower signal; lower right, proteolytic sites based on protein mass spectrometry and Edman sequencing data. Uncropped gels are shown in Supplementary Fig. 8.

We then sought for the common C-terminal fragment of this cleavage event to identify the exact cleavage site. Using our in-house C-terminal-specific tau antibodies, we immunoprecipitated and identified an additional C-terminal fragment of tau that is present at low levels in all samples, with an apparent molecular weight of 14 kDa (C-LMW4, Fig. 2d). This common C-terminal fragment is distinct in size from C-LMW1-3 from late stage AD brains. We isolated both N-LMWs and C-LMW4 from control lysate for tandem protein mass spectrometry. Fragments were immunopurified with N-terminal specific (N-ter, in house) or C-terminal specific antibodies (C-ter-1, in-house) and resolved by polyacrylamide gel electrophoresis, and N-LMWs, C-LMW4 as well as a full-length tau band were excised, tryptic-digested and subjected to LC/MS/MS (Fig. 3a). The distribution of tryptic and semi-tryptic peptides from N- and C-fragments along 2N4R tau protein sequence is visualized by peptide pileup plot in Fig. 3b. A clear demarcation at around AA320 was observed between N-LMWs and C-LMW4 signals, and a peak of semi-tryptic peptides (AA323-340) specific to the C-LMW4 was found at the C-terminal side this boundary. The majority of semi-tryptic peptides in the C-terminal fragment mapped to this region, which contained few peptides from the N-LMWs and full-length tau. The two major semi-tryptic peptides are S324-K340 and N327-K340, indicating cleavage sites at G323-S324 and G326-N327 (Fig. 3c). The vast majority of peptides from N-LMWs mapped in between AA7-AA317 with no prominent semi-tryptic peptides. As AA318 and AA321 are both lysine residues, the semi-tryptic peptides from N-terminal fragments ending at G323 and G326 are likely too short to be detected in MS/MS.

To further confirm the N-terminal sequences of immunopurified C-LMW4, we performed Edman degradation protein sequencing. We purified ~1 μg of C-LMW4 from control frontal cortex lysate by immunoprecipitation. As shown in Fig. 3d, two N-termini were identified and these corresponded to the two major semi-tryptic peptides from protein mass spectrometry. These results strongly support endogenous proteolytic cleavages of tau at G323/S324 and G326/N327 in the normal human cortex.

### Characterization of C-terminal tau fragments specific to late stage AD

**Figure 4:**
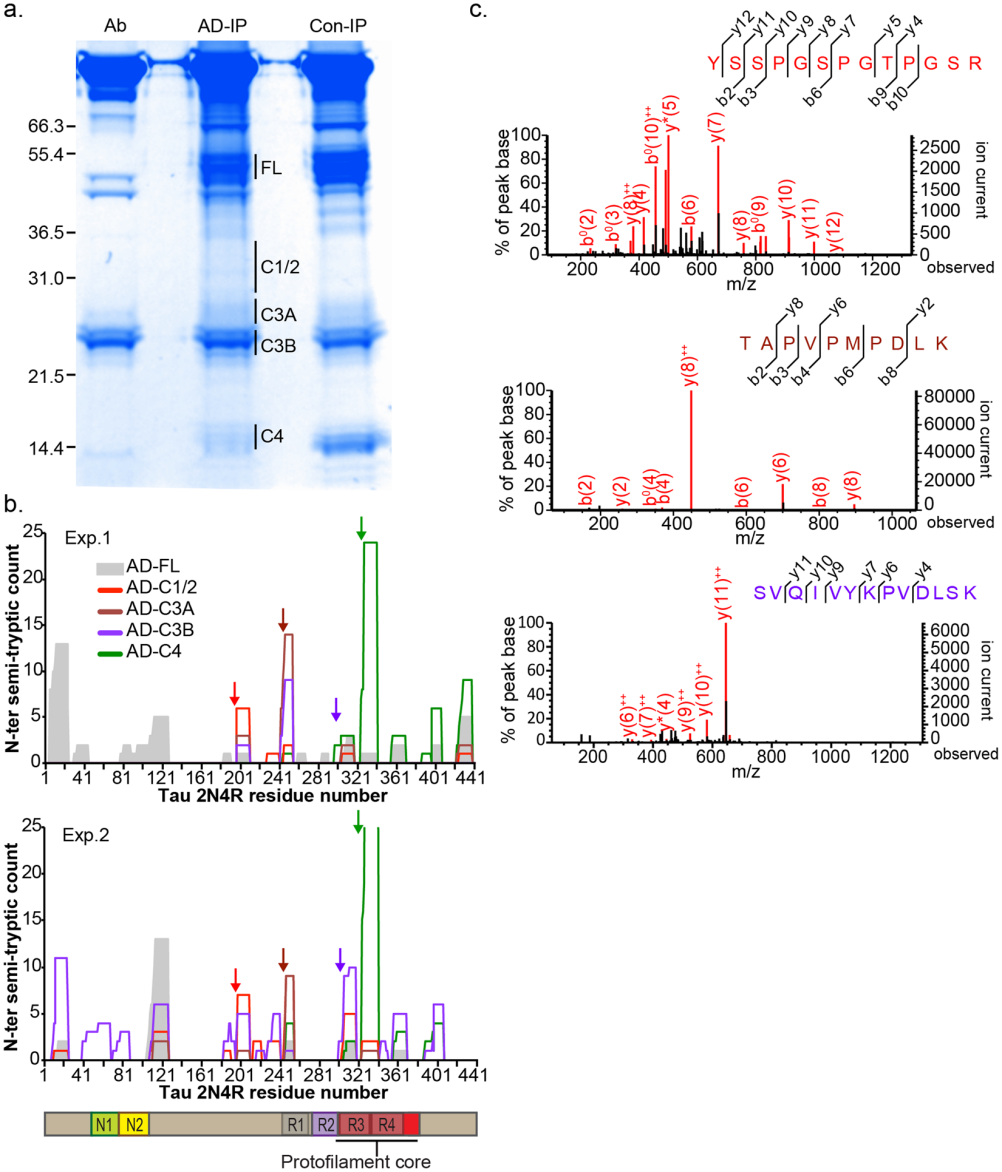
Characterization of pathological tau fragmentation. (**a**) Immuno-purification of C-terminal tau fragments for LC/MS/MS from pooled AD (Braak stages V and VI) fusiform gyrus lysate using C-ter-1 antibody. Protein bands were resolved in non-reducing SDS-PAGE. Ab, C-ter-1 antibody only, AD-IP, IP from pooled AD samples, Con-IP, IP from control frontal lysate. (**b)** Semitryptic peptides with non-tryptic site at the N-terminus from the C-LMWs mapped onto 2N4R tau sequence. 2 biological repeats using different pools of patient samples. (**c)** Protein mass spectrometry traces of representative semi-tryptic peptides enriched in AD V/VI C-terminal fragments. Arrows, non-tryptic digestion sites. Uncropped gels are shown in Supplementary Fig. 8.

We then used LC/MS/MS to identify the cleavage events that generate C-LMW1-3. The C-terminal fragments were immunoprecipitated from fusiform gyrus lysates pooled from 8 (pilot experiment), 19 (experiment #1) and 22 (experiment #2) AD patients. Samples in the pilot experiment did not overlap with the other experiments; those in experiments #1 and #2 are partially overlapping. Immunoprecipitated tau was resolved by SDS-PAGE in non-reducing conditions to minimize IgG light chain signal in the ~25 kDa region (Fig. 4a). IP from normal frontal lysate was performed in parallel as negative controls for C-LMW1-3. Bands with apparent molecular weights corresponding to C-LMW1, 2, 3 and 4 and full-length tau were excised and analyzed as described above and in Methods. Semi-tryptic peptides with N-terminal non-tryptic sites are mapped back to 2N4R protein sequence. In all three experiments, the 14 kDa C4 band from AD brain lysates yielded the same two semi-tryptic peptides as C-LMW4 from control brain lysate (Fig. 4b, green lines and green arrows). In C-LMW1, 2 and 3, we identified additional semi-tryptic fragments N-terminal to G323 that are absent from full-length tau (Fig. 4b, red, brown and purple lines). Based on apparent sizes and peptide counts, three regions were identified as likely cleavage sites for each fragment: G196, L243-T245 and G303-G304 (representative MS traces in Fig. 4c). Each of these semitryptic peptides were also found in the pilot experiment, although the peptide counts for the entire experiment were low due to lower protein input.

### *In vitro* calpain 1 digestion of recombinant tau recapitulates *in vivo* cleavage

We set forth to identify the endogenous protease(s) mediating the G323/G326 cleavages in the normal cortex. Based on the primary sequence, the cleavage sites are unlikely to be generated by caspases or legumain (asparagine endopeptidase), two of the recently reported tau proteases^15,23^. We hypothesized that intracellular tau should be readily accessible to its endogenous protease, based on the substantial proportions of fragments present in the brain, narrowing down our search to cytosolic proteases. Starting with all known human proteases in the Degradome database^43^, we screened for protease candidates by excluding extracellular, lysosomal, mitochondrial and proteasome peptidases, as well as proteases with established substrate specificity inconsistent with the cleavage sites and those showing no expression in the brain. Of the 23 remaining proteases, 10 belong to the calpain family (Supplementary Table 1). Previous studies showed that tau is a calpain substrate albeit with different cleavage sites^28,44–48^: we tested if the G323/G326 sites observed in normal human cortex could be recapitulated *in vitro* with calpain 1. Under limited proteolysis, we observed a major N-terminal fragment of 1N3R tau at around 40 kDa, comigrating with endogenous LMW2 (1N3R fragment, Supplementary Fig. 2a left). Similarly, limited digest by calpain 1 generated a 45 kDa N-terminal fragment from 2N4R tau, the size of which is consistent with the endogenous 2N4R fragment (see below). The C-terminal fragments from both isoforms have apparent molecular weights similar to the 14 kDa endogenous fragment (Supplementary Fig. 2a right and below). We also isolated the 40 kDa and 14 kDa bands from 1N3R tau calpain 1 digest for tandem mass spectrometry. The distribution of peptides of these two fragments along the 1N3R protein sequence is similar to that of the endogenous N-LMWs and C-LMW4 (Supplementary Fig. 2b). In the 14 kDa band, we observed semi-tryptic peptides starting at S324 and N327 (Supplementary Fig. 2c), which are identical to those in C-LMW4 from brain. To confirm that the *in vitro* cleavage sites are the same as the endogenous ones, we quantified the levels of the two 14 kDa fragments (S324-L441 and N327-L441) in the calpain 1 reaction by middle-down protein mass spectrometry. Both fragments were detected upon calpain 1 digestion but not in the input recombinant tau (Supplementary Fig. 2d). These results support a calpain-mediated mechanism of normal tau cleavage.

**Figure 5:**
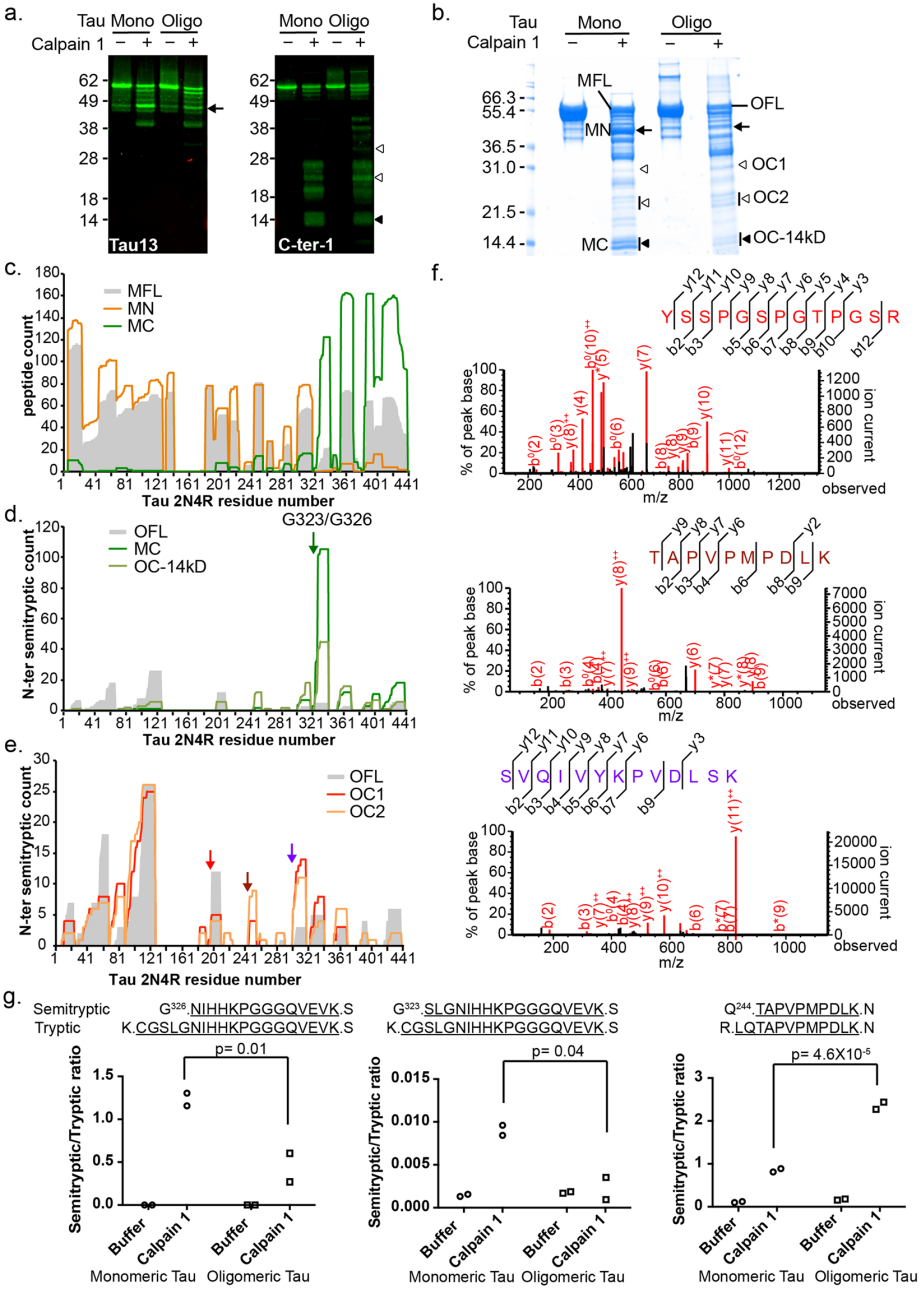
Calpain 1 digest recapitulates *in vivo* tau cleavage. (**a)** N-ter and C-ter tau immunoblots showing monomeric and oligomeric tau differentially cleaved by calpain 1. Arrow, N-terminal fragment with reduced signal in oligomeric tau digest; solid arrowhead, C-terminal fragment with reduced signal in oligomeric tau digest; open arrowheads, C-terminal fragments similar in size to *in vivo* AD-enriched fragments and with increased signal in oligomeric tau digest. (**b**) SafeBlue staining of calpain 1-digested monomeric and oligomeric tau 2N4R for LC/MS/MS identification of tau oligomer-specific calpain 1 cleavage sites. Bands denoted by arrows, solid arrowheads and open arrowheads correspond in molecular weight to those in **(a)**. MFL, full length tau from monomeric digest, MN, major N-terminal fragment in monomeric tau digest, MC, 14 kDa C-terminal fragment in monomeric tau digest, OFL, full length tau from oligomeric digest, OC1 and OC2, group of C-terminal fragments from oligomeric digest, OC-14kD, 14 kDa C-terminal fragment from oligomeric digest. (**c**) Tryptic and semitryptic peptides of MN and MC fragments from monomeric tau digest mapped to 2N4R sequence. (**d**) N-terminal semitryptic fragments of MC and OC-14kD mapped to 2N4R sequence, full-length oligomeric tau included as control (OFL). (**e**) N-terminal semitryptic fragments of OC1 and OC2 mapped to 2N4R sequence, OFL included as control. (**f**) Protein mass spectrometry traces of representative semi-tryptic peptides identified from OC1 and OC2 fragments. (**g**) Comparison of calpain 1 cleavage of monomeric and oligomeric tau 2N4R at G323, G326 and Q244 by targeted mass spectrometry quantification (MRM). The levels of semitryptic peptides were normalized to respective tryptic peptides shown above each plot (N = 2). Uncropped gels are shown in Supplementary Fig. 8.

As described earlier, the levels of C-LMW1-3 correlated tightly with the HMW tau smear, suggesting that these AD-enriched C-LMWs may be derived from tau aggregates, in which G323 and G326 are buried within the pronase-resistant protofilament core^49^ that are likely inaccessible to proteases including calpain. To test this hypothesis, we analyzed calpain 1 digestion of *in vitro* oligomerized tau. Oligomeric tau was prepared from recombinant 2N4R tau by *in vitro* aggregation (Methods), and the resulting preparation is a mixture of monomeric and oligomeric tau (Supplementary Fig. 3). After calpain 1 limited digestion, we observed differential fragmentation patterns in oligomeric versus monomeric tau in both the N-terminal and C-terminal specific immunoblots; specifically, a reduction of the 45kDa N-terminal and 14 kDa C-terminal fragments, as well as an increase in larger C-terminal fragments, including a 24 kDa band (Fig. 5a,b) with oligomeric tau as substrate. Using protein mass spectrometry, we observed the peptide distribution of MN and MC bands, main products of the monomeric digest, to be consistent with these as complementary fragments produced by a cleavage site at around G323/G326 (Fig. 5c). Semitryptic peptides of the 14 kDa bands from both monomeric and oligomeric digests (MC and OC-14kD) confirmed the same cleavage sites (Fig. 5d). There appear to be no difference in calpain 1 site recognition between tau isoforms 1N3R and 2N4R at the monomeric state. LC/MS/MS of the cleavage products enriched in oligomeric tau digest revealed multiple non-tryptic N-termini that were shared with AD-enriched C-LMWs (Fig. 5e,f). To ascertain the shift in cleavage site preference upon tau aggregation, we performed quantitative analysis of semitryptic fragments corresponding to each cleavage site using targeted protein mass spectrometry (MRM). Cleavages at G323 and G326 were found to be significantly higher (2.5 and 3-fold, Fig. 5g) in monomeric versus oligomeric tau calpain 1 digests. In contrast, cleavages at Q244 and T245 are enriched in oligomeric tau digest by 2.8 and 1.7-fold (Fig. 5g, Supplementary Table 2). These cleavages were specific to calpain 1 treatment. Cleavage at G196 yielded readings in low picogram ranges with no difference between monomeric and oligomeric digests, and cleavage at G304 was not detectable in this assay (Supplementary Table 2). None of the tryptic peptides measured showed any difference between monomeric and oligomeric digest, or between buffer controls and calpain 1 digests. Together these *in vitro* results support that calpain family proteases mediate both the normal (G323 and G326) and at least one set of AD-enriched cleavages (Q244 and T245) of tau, and the difference in cleavage site selection is dependent on substrate conformation instead of a change in protease specificity.

## Discussion

Endogenous proteolytic fragments of tau have been consistently observed across numerous studies. A number of proteolytic sites were identified in *in vitro* systems and transgenic murine models and proposed to generate fragments similar in apparent molecular weight to those found in human brain lysates^15,23^. Specific C-terminal and N-terminal truncation sites have been designated to fragments in human brain tissue; however, it is unclear whether the main fragmentation pattern observed in brain lysates are products of these cleavage events. To our knowledge, this is the first comprehensive study to directly define the main fragments of tau found in the human cortex.

Using post-mortem human cortical tissue, we uncovered two novel cleavage sites within the 3^rd^ microtubule-binding repeat (MTBR3) that generates major species of tau fragments and are shared among all 6 isoforms of tau in the human brain. The common fragmentation pattern across all patient samples, including control tissue of two separate cortical regions, and the relative abundance of the fragments suggest that these cleavage events are part of normal tau protein processing. Importantly, the endogenous levels of the fragments produced by these cleavage events were sufficient for identification by Edman degradation, strongly supporting that endogenous tau is precisely and predominantly processed at these sites.

The recent Cryo-EM study of patient-derived tau tangles resolved the structure of paired helical filaments (PHF) and straight filaments to atomic level and defined the protofilament core, which spans V306-F378, MTBR3-R4^49^. Intriguingly, the normal tau cleavage sites we identified are located at the center of the core structure protein sequence, and G323 at the P1 location comprises one of the glycines required for β-helix formation. The structural importance of this segment is consistent with an earlier study using solid-state nuclear magnetic resonance spectroscopy, in which V306-S324 was identified as the rigid core of PHFs, and a large kink at C322-G323 was inferred^50^. Furthermore, a short tau fragment of 43 residues (N265-E342ΔR2) comprised mainly of MTBR3 and spanning the normal tau cleavage site has been shown to self-assemble into filaments and serve as nucleating seeds for *in vitro* PHF formation^51^. Two FTDP-17 causal tau mutant proteins, V337M and R406W, were shown to be more resistant to calpain-1 digestion than WT or P301L tau^52^, hinting at a potential role of calpain in tau reduction. Overall, the structural information of this region suggests tau cleavage at G323/G326 is likely to interfere with PHF formation, in line with our observation that normal tau fragmentation decreases with increasing severity in tau pathology.

**Figure 6:**
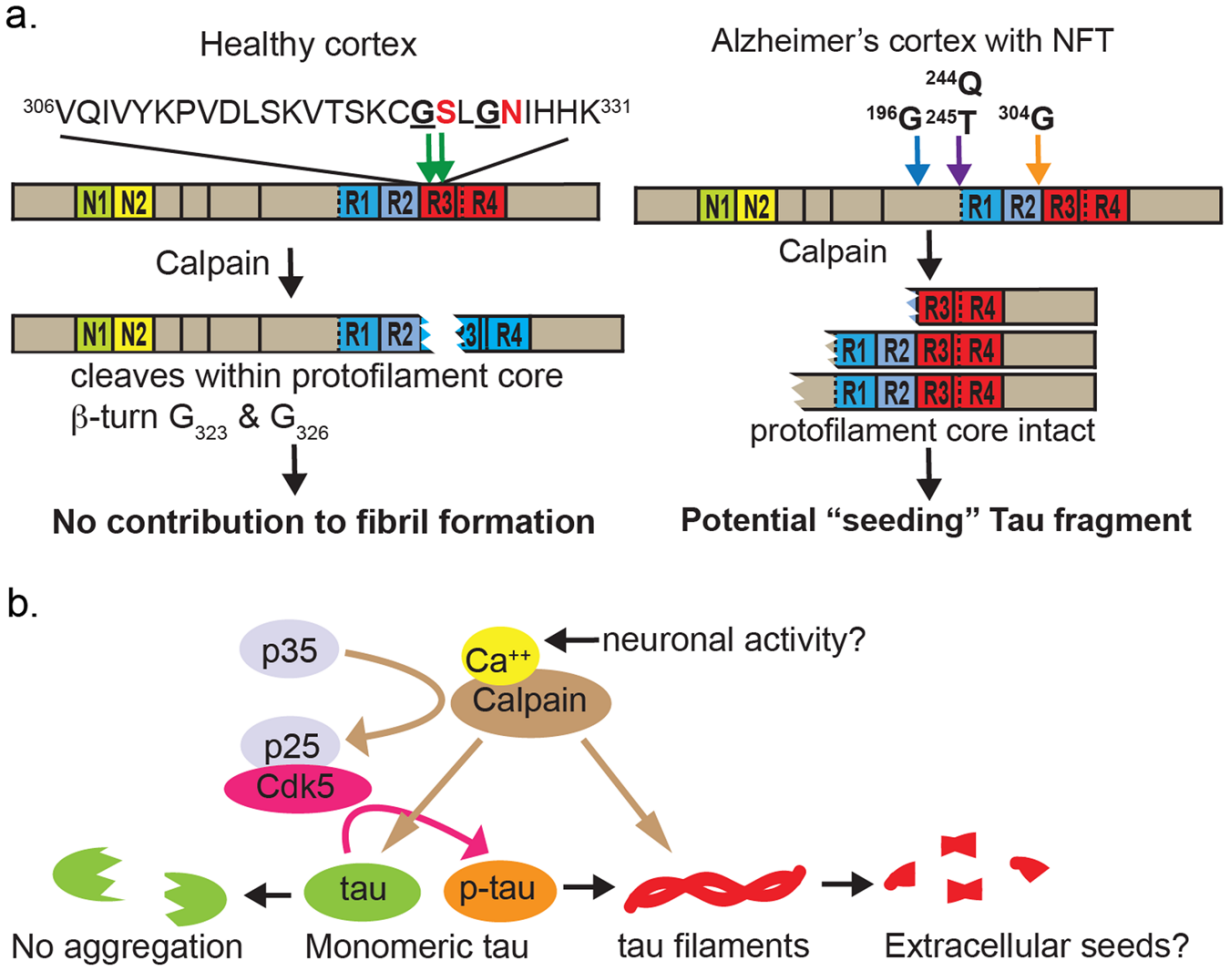
Schematics of tau fragmentation pathway in cortical maintenance and Alzheimer’s disease. (**a**) Tau is differentially fragmented in healthy and Alzheimer’s disease cortices. The endogenous P1 cleavage sites identified in this study are highlighted in red in the peptide sequence. The R3 and R4 boxes comprising the fibril core are filled in red. (**b**) Hypothesis of calpain-mediated tau maintenance and pathology propagation.

We also identified multiple cleavage events enriched in late stage AD brains. All of these cleavage sites are N-terminal to V306 and would leave the protofilament core intact. We showed that both the normal and AD-enriched tau cleavage could be reproduced by calpain-1 digestion *in vitro*, and the selection of the proteolytic site is dependent on substrate conformation: oligomerized tau preferentially cleaved at sites specific to late-stage AD, and monomeric tau at the normal sites. The difference is consistent with the observation that the pronase-resistant core of protofilaments spans MTBR3-R4, the region presumably inaccessible to proteases after oligomerization^49^. One AD-enriched tau cleavage region (Q244/T245), the calpain 1 cleavage site favored in oligomeric tau *in vitro*, is in close vicinity to the previously reported R242 truncation^16^, which generates a 24 kDa band that increases with age in mice overexpressing human tau, and has higher aggregation and seeding activity than full length tau *in vitro*^16^. R242 is however a tryptic site and from our analysis, the 24 kDa band would instead be consistent with a calpain 1-mediated Q244 cleavage of aggregated tau, which also increases with age. From these observations we propose that the normal fragmentation pathway comprise part of the tau quality control mechanism in counteracting filament formation or growth; however, once the filaments have formed the tau protease(s) could no longer access the optimal residues for preventing β-helix formation and cleaves elsewhere (Fig. 6a). Consistent with our hypothesis, HMW tau in AD brains is recently reported to be dominantly truncated in the N-terminal region, whereas D421 cleavage correlated poorly with HMW tau levels^17^. The sub-optimal cleavage events produce C-terminal fragments that presumably retain the structural features of the pronase-resistant protofilament core^49^.

We show here that calpain 1 can cut precisely at the identified sites on unmodified tau *in vitro*. While not precluding the possibility of other tau proteases involved *in vivo*, our results suggest the endogenous protease in question likely belongs to the calpain family. Indeed, over the past decades several reports have indicated μ-calpain as a tau protease^27,53^ that generates a 17kDa N-terminal to mid-domain fragment with neurotoxicity^28,48^. Moreover, calpain has a well-established role in regulating tau phosphorylation via processing of p35 to p25, the latter of which acts as a stronger neuronal-specific activator of tau kinases CDK5^54^ and GSK-3β^55^. Stemming from these findings and promising preclinical data, calpain inhibitors have been explored as a potential treatment for AD^56^. Our results add complexity to the role of calpain in tau regulation: depending on the downstream target and excision site, calpain activity may either promote (eg. activating tau kinase) or inhibit (eg. normal tau fragmentation) fibril formation (Fig. 6b). The multiple calpains, regulators and inhibitors in CNS neurons, along with their calcium-dependent nature coupled to activity-dependent calcium fluctuations provides many potential levels of control mechanisms that awaits further investigation.

Elevated tau levels in the CSF are among the few core fluid biomarkers for Alzheimer’s disease^57^. Current immunoassays for both total and phospho-tau, where epitope information is available, predominantly target the mid-domain (AA150-250). The vast majority of tau species in the CSF are, however, highly fragmented beyond AA225^41,42,58^ and consequently the clinical measurement of CSF tau is limited to the detectable fragments, mostly mid-domain, while potential changes in the C-terminal fragment during disease progression is unclear. Future work is needed to determine whether the marked difference of C-LMWs between early and late Braak stages is reflected in the CSF and their potential as biomarker candidates.

## Methods

### Ethics statement

All tissue donors or their legal representatives signed an Institutional Review Board-approved informed consent form allowing both clinical assessments during life and brain donation after death and its approval includes the collection of human organs at autopsy for unlimited number of research studies. The name of the IRB is Western Institutional Review Board (1019 39^th^ Ave SE #120, Puyallup, Washington, USA). The title of the IRB approved study is “Brain and Body Donation Program” and autopsies are performed by the Banner Sun Health Institute. A separate section requests to allow or disallow DNA isolation, storage and genetic testing. All procedures performed in studies involving human participants were in accordance with the ethical standards of the institutional and/or national research committee and with the 1964 Helsinki declaration and its later amendments or comparable ethical standards.

All vertebrate animal experiments were performed in accordance with Swiss legislation and the European Community Council directive (86/609/EEC) for the care and use of laboratory animals, and were approved by the Veterinarian Office of the canton of Vaud, and the local animal care ethics committee. We confirm that all experiments were performed in accordance with the relevant guidelines and regulations.

### Patient samples

Frozen post-mortem fusiform gyrus and pathology/clinical data of 35 control, 46 AD Braak III/IV and 46 AD Braak V/VI de-identified patients and control frozen frontal cortex blocks were obtained from the Banner Sun Health Research Institute Brain and Body Donation Program^59^. Braak staging was performed on 40 μm thick sections with the Gallyas stain, as originally described in^60^. All samples were analyzed for N-terminal fragments. A small number of samples (3 control and 3 AD III/IV) were depleted and not included in the C-terminal fragment analysis.

Fusiform gyrus cryosections were homogenized using TissueLyzer II (Qiagen) in ice-cold RIPA buffer (Sigma-R0278) containing protease inhibitors (Complete mini, Roche). Frozen blocks of control frontal tissue were ground with pre-chilled Cryo-Grinder in LN2 before homogenization with TissueLyzer. Homogenized samples were centrifuged at 14,000 g for 20 min at 4 °C. Protein content in the supernatants was determined using Pierce BCA protein assay kit before aliquoting and storage at −80 °C.

### Immunoblotting and immunoprecipitation

For immunoblotting, samples were heated at 70 C in 1 × sample loading buffer containing reducing agents for 5 min, separated on NuPAGE Bis-Tris gels in MOPS or MES buffer (Life Technologies) and transferred to PVDF (iBlot2, Life Technologies). Primary antibodies are: mouse Tau13 (1:2000, MBL JM-3453-100), mouse HT7 (1:500 Thermo Fisher MN1000), mouse anti-RD3 (1:500, Millipore 8E6/C11 #05-803), rabbit anti-human Tau (1:1000, Dako A0024), rabbit anti-4R-tau (1:2000, CosmoBio TIP-4RT-P01), mouse α-tubulin (1:2000 Cell Signaling Technology #3873), rabbit β-actin (1:2000, Cell Signaling Technology 13E5 #4970), and antibodies developed with AC Immune SA (below). The protein bands were visualized with IRDye-conjugated secondary antibodies using the Odyssey Imaging System (LI-COR Biosciences).

For immunoprecipitation, protein G or protein A Dynabeads (Thermo Fisher) were prebound with specified antibodies or normal mouse IgG (Millipore #12-371) or purified human IgG (R&D systems 1-001-A) before incubation with brain lysates at 4 °C. DynaBeads were then extensively washed in RIPA buffer with protease inhibitors and eluted by heating in 1X LDS sample buffer with or without reducing reagents (Thermo Fisher).

### Recombinant tau and oligomeric tau

Codon-optimized sequence of human tau 2N4R AA2-441 was cloned into a modified pGEX4T-1 vector (GE Healthcare) containing a N-terminal His-tag, expressed in Escherichia coli and affinity-purified using a Ni-NTA column followed by gel filtration using a Superdex 200 column (GE Healthcare). The His-tag was cleaved using TEV protease, and recombinant tau was purified by cation exchange HiTrap SP HP column (GE Healthcare). The purity and identity of the isolated protein was confirmed by SDS-PAGE and mass spectrometry. The purified tau protein was oligomerized using 75 μM arachidonic acid (Cayman Chemicals) and 18 kDa Heparin (Sigma-Aldrich), at equimolar concentration with protein sample, in 20 mM BES, 25 mM NaCl, pH 7.4 at 37 °C for 3 days. Oligomerization was confirmed by thioflavin T fluorescence assay and dynamic light scattering.

### Antibody generation

Mice were vaccinated with either oligomeric tau or a liposomal vaccine containing full-length human 2N4R tau (flTau; SignalChem). Adjuvant used was Ribi Adjuvant System (Sigma-Aldrich) at 50% v/v, or a combination of CpG single-stranded synthetic DNA (Microsynth) and aluminum hydroxide (Brenntag). Liposomal vaccines were produced as described in^61^, with the following modifications: flTau was reduced at a TCEP (Sigma-Aldrich):protein molar ratio of 100:1 for 30 min at 23 °C and coupled to DSPE-PEG(2000)maleimide lipid (Avanti Polar Lipids) at a lipid/protein molar ratio of 30:1 at 23 °C for 4 h. The product was incubated with preformed liposomes for 15 h at 37 °C. Liposomes were further subjected to ultrafiltration and diafiltration in PBS pH 7.4, sterile filtered through 0.2 μm polyethersulfone membrane, and stored at 4 °C.

Female C57BL/6JOlaHsd, BALB/c OlaHsd (Harlan), or tau knock-out mice (B6.129-Mapttm1Hnd/J; Jackson Laboratory) were vaccinated by subcutaneous (s.c.) injection, or by intraperitoneal and hock administrations. Before hybridoma fusion, three booster s.c. injections of adjuvant-free vaccine were administered daily. Hybridomas were selected using ELISA or Luminex multiplex target assays. Antibodies were purified by protein G affinity chromatography or by thiophilic adsorption and ammonium sulfate precipitation.

### Immunoblot quantification

IRDye fluorescence signal was quantified in ImageStudio (LI-COR Biosciences). Full-length bands from N-ter and C-ter immunoblots were captured with 4 boxes corresponding to MW ranges of recombinant 2N4R, 2N3R + 1N4R, 1N3R + 0N4R and 0N3R. We chose full-length + fragments instead of entire lanes to avoid potential non-specific signal, and test quantifications in a subset of samples did not yield substantial differences between the two methods. Fragment bands were individually quantified. HMW smear signal was quantified with one box above the 70 kDa marker. The percentages of individual N-LMWs and their sums against total N-ter tau signal (sum of full-length and 6 fragments) were analyzed for correlation with patient groups, histopathology and clinical features. The percentages of individual C-LMW bands and their sums against C-ter tau signal (sum of full-length, HMW smear and 3 fragments) were analyzed similarly. The HMW smear was not prominent in N-ter immunoblots and was thus not included. The linear range was determined with serial dilutions of recombinant 2N4R (not shown).

### Tandem mass spectrometry (LC/MS/MS)

SDS-PAGE bands were excised, washed in 50 mM ammonium bicarbonate in 50∶50 acetonitrile:water for 20 min, dehydrated with acetonitrile and trypsin-digested overnight (Promega) at 37 °C. Peptides were extracted in 50∶50 v/v acetonitrile: 1% formic acid (Sigma) for 30 min followed by equal volume acetonitrile. Pooled extractions were reconstituted in 2% acetonitrile: 0.1% formic acid and injected onto a 75 µm × 100 mm column (BEH, 1.7 micron, Waters Corp) using a NanoAcquity UPLC (Waters Corp). A gradient from 98% solvent A (0.1% formic acid) to 80% solvent B (acetonitrile + 0.08% formic acid) was applied over 40 min. Samples were analyzed via nanospray ionization into a hybrid LTQ-Orbitrap Elite mass spectrometer (Thermo Fisher). Data was collected in data dependent mode with the parent ion analyzed in the FTMS and the top 15 most abundant ions selected for fragmentation and analysis in the LTQ. MS/MS data was analyzed using the Mascot algorithm (Matrix Sciences).

### Peptide Pileup Plots

Using a custom R script, each unique mass-spec peptide sequence (such as “K.AKTDHGAEIVY#KS#PVVSGDTSPR.H”) was parsed into flanking amino acids (“K” and “H”) ignoring post-translational modifications. Exact matches of the full peptide, along with the flanking amino acids, in the tau 2N4R or 1N3R sequence were identified. Peptides not matching exactly along with flanking amino acids, if any, were discarded, as were peptides, if any, that occurred more than once in the protein. Flanking amino acids were removed, and the number of peptides aligned to each position of the full-length protein sequence was counted and plotted.

### Middle down MS and MRM analysis

Recombinant tau 1N3R and 2N4R (rPeptide) at 0.4 mg/ml were digested with 10, 2.4 or 1 U/ml natural human calpain 1 (Abcam AB91019) in 50 mM Tris pH 7.5, 100 mM NaCl, 2 mM dithiothreitol and 3 mM CaCl_2_ at 30 °C for specified min and stopped with addition of loading buffer for SDS-PAGE or equal volume of 8 M GuHCL for MS.

For middle down MS, samples were analyzed in data dependent acquisition mode (DDA) using a Thermo Q Exactive HF mass spectrometer. The MS^1^ scans were acquired at a resolution of 60,000 in the scan range of 300–1500da. The top ten MS^2^ spectra were acquired at a resolution of 15,000 using an isolation window of 1.5da and a dynamic exclusion window of 10 s. MS/MS data was analyzed using the Mascot algorithm. Sample introduction and separation was performed on an Eksigent ekspert NanoLC 425. Samples were loaded in solvent A onto a Zorbax SB300 C18 5μm trap column (Agilent) and a gradient from 98% solvent A to 32% solvent B’ (acetonitrile + 0.1% formic acid) was applied over 90 min on a Zorbax SB300 C18 3.5μm column (Agilent).

For MRM, we selected peptides for quantitation from prior DDA data. Sequences were input into Skyline (U. Washington) and optimization was performed on a 6500 mass spectrometer (Sciex) coupled to a M3 HPLC (Eksigent). Standard curves were generated in triplicate with synthetic peptides (Elim Biosciences) at >95% purity (Supplementary Table 2) with iRT (Biognosys) standards.

Samples were reduced in 5 mM dithiothreitol (Pierce), 6 M GuHCl and 0.1 M Tris pH 8.0 at 37 C for 1 h, alkylated in 10 mM iodoacetimide (Pierce) at 24 C for 40 min, digested with trypsin/LysC (Promega) for 14 h at 37 C, acidified in 1.0% formic acid (Fluka) and desalted using an Agilent AssayMap Bravo with RPW cartridges. Eluted samples were dried, reconstituted in 2% acetonitrile with 0.1% formic acid with iRT internal standards and loaded in duplicate in solvent A onto a Zorbax SB300 C18 5μm trap column (Agilent). A gradient from 98% solvent A to 32% solvent B’ was applied over 25 min on a Zorbax SB300 C18 3.5μm column (Agilent). Data was analyzed and quantitated in Skyline.

### Edman degradation

Proteins resuspended in SDS buffer +20 mM DTT were separated on 4–20%, wet-transferred to PVDF membranes in NuPAGE transfer buffer and briefly stained with 0.1% Coomassie Blue R250. The bands of interest were excised and subjected to Edman sequencing analysis using the Applied Biosystems Procise Sequencer Model 494HT. Peptide sequence was analyzed with SequencePro 2.1 (Applied Biosystems) and manually validated.

## Electronic supplementary material

Supplementary Figures and Tables

## Data Availability

In accordance with the informed consent given by the study participants, the anonymized datasets generated during and/or analyzed in this study are available from the corresponding author on reasonable request.
