# Supplementary material for: Calpain-mediated tau fragmentation is altered in Alzheimer’s disease progression
Source: Sci Rep. 2018 Nov 13;8:16725. doi: 10.1038/s41598-018-35130-y (PMC6233188; doi:10.1038/s41598-018-35130-y)
Supplement: Supplementary file 1 — Supplementary Figures and Tables [file 41598_2018_35130_MOESM1_ESM.pdf]

Calpain-mediated tau fragmentation is altered in Alzheimer's disease progression

Hsu-Hsin Chen, Peter Liu, Paul Auger, Seung-Hye Lee, Oskar Adolfsson,

Lorianne Rey-Bellet, Julien Lafrance-Vanasse, Brad A. Friedman, Maria Pihlgren,

Andreas Muhs, Andrea Pfeifer, James Ernst, Gai Ayalon, Kristin R. Wildsmith,

Thomas G. Beach, Marcel P. van der Brug

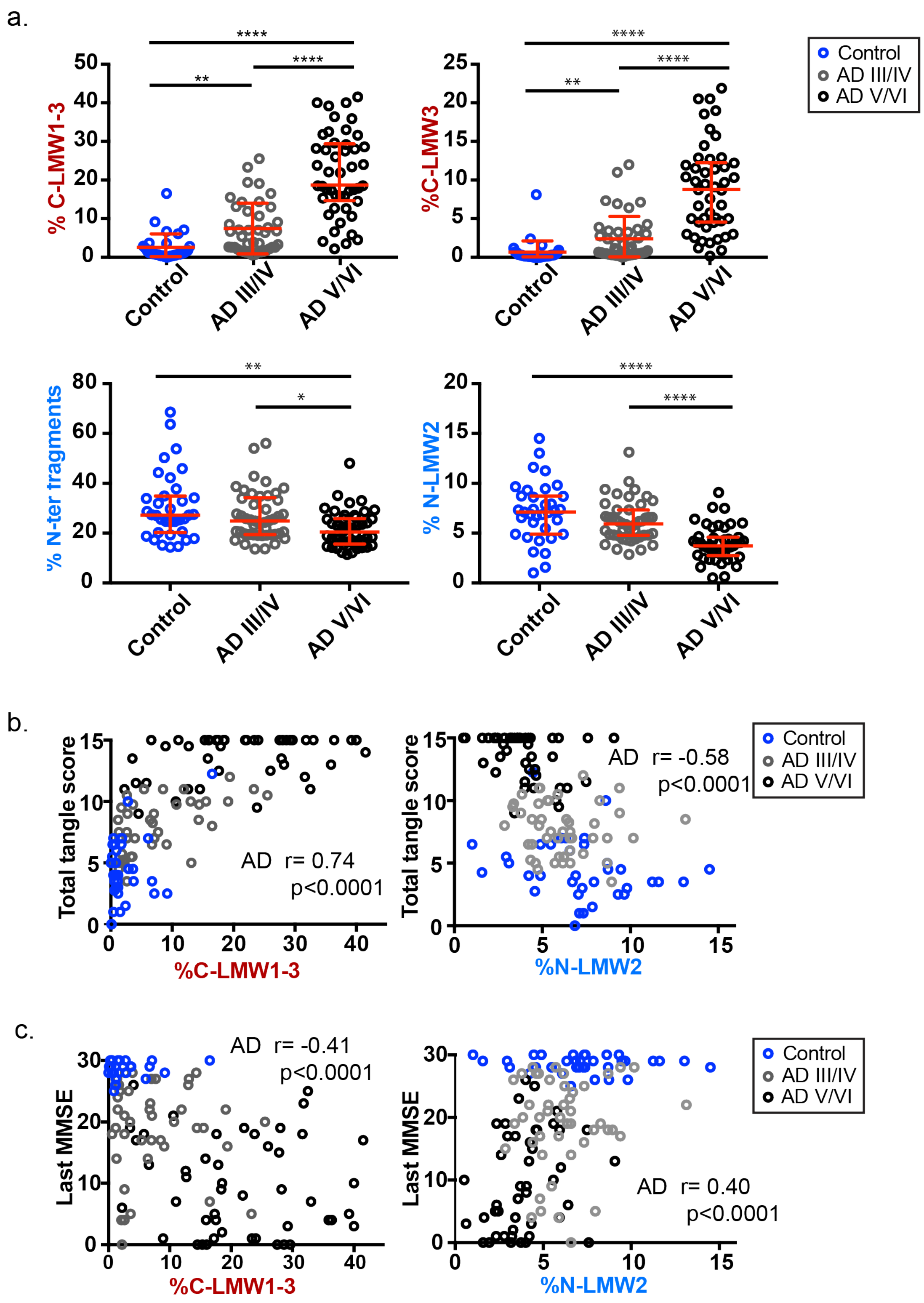
a.
% C-LMW1-3
%C-LMW3
% N-ter fragments
% N-LMW2
Control
AD III/IV
AD V/VI
b.
Total tangle score
%C-LMW1-3
%N-LMW2
AD r= 0.74
p<0.0001
AD r= -0.58
p<0.0001
c.
Last MMSE
AD r= -0.41
p<0.0001
AD r= 0.40
p<0.0001

Supplementary Figure 1. Changes in tau fragment composition correlates with clinical and histopathological parameters of progression. **a.** Percentages of C- and N-tau fragments across patient groups. C-terminal fragments: Control, N=32, AD III/IV, N=43, AD V/VI, N= 46; N-terminal fragments: Control, N=35, AD III/IV, N=46, AD V/VI, N=46. The discrepancy in N is due to deletion of a small number of samples. C-LMW3 and N-LMW2 showed the lowest p values in Dunn's multiple comparison test, * $p<0.05$, ** $p<0.01$, *** $p<0.001$, **** $p<0.0001$. Bars represent median with interquartile range. **b.** Relationship between fragment abundance and total tangle score (sum of histopathological scores from 5 brain areas: frontal cortex, parietal cortex, temporal cortex, hippocampus and entorhinal cortex). %C-LMW1-3 showed strong positive correlation with total tangle score and %N-LMW2 showed negative correlation. **c.** Relationship between fragment abundance and cognition. %C-LMW1-3 showed negative correlation with the last MMSE score taken (Spearman's rank correlation). N-LMW2 showed positive correlation with the final cognitive score. ρ and p values of AD patients are shown on graphs. Including control patients in Spearman's correlation does not change the direction of correlation: C-LMWs vs tangle score, $\rho=-0.80$, $p<0.0001$ C-LMWs vs last MMSE, $\rho=-0.63$, $p<0.0001$. N-LMW2 vs tangle score, $\rho=-0.58$, $p<0.0001$ N-LMW2 vs last MMSE, $\rho=0.45$, $p<0.0001$.

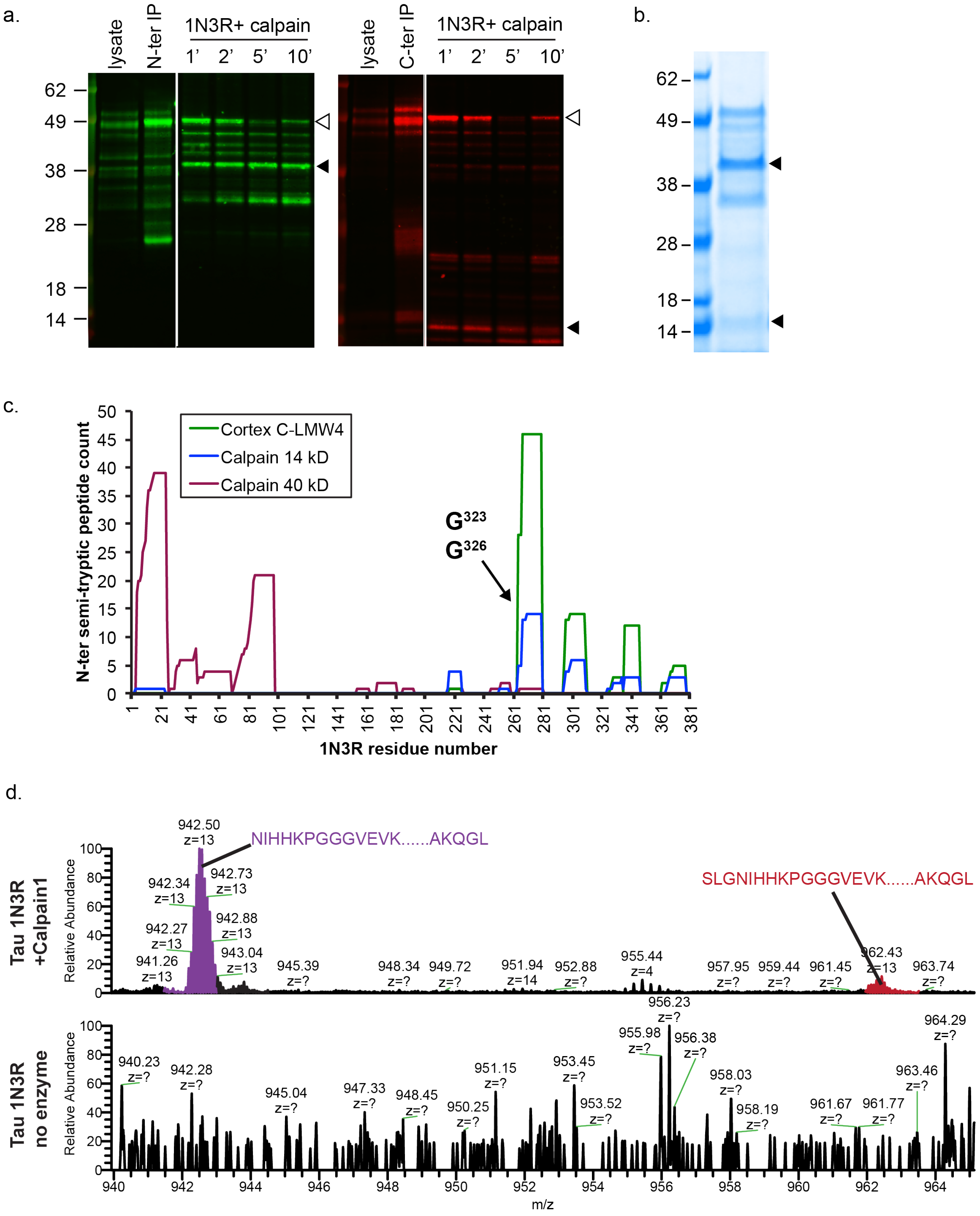

Supplementary Figure 2. Identification of calpain cleavage sites in tau 1N3R isoform. **a.** N-ter and C-ter tau immunoblots of *in vitro* calpain-1 digest of recombinant 1N3R tau protein. Lysate, 1/10 of control frontal cortex lysate input

used in IP; N-ter IP and C-ter IP, IP from 100 μg of control frontal cortex lysate; 1', 2' 5' and 10', recombinant 1N3R tau digested with calpain-1 for respective minutes at 37°C. Open arrowheads, full length 1N3R tau; black arrowheads, N- and C-terminal fragments similar in apparent molecular weight to N-LMW2 and C-LMW4. **b**. SafeBlue staining of calpain-digested tau 1N3R for LC/MS/MS identification of calpain-1 *in vitro* cleavage sites. **c.** N-terminal semitryptic peptides of *in vitro* fragments compared to the endogenous C-terminal fragment mapped to tau 1N3R protein sequence. The cleavage site residues are denoted with 2N4R residue numbers. **d.** Middle-down protein mass spectrometry quantification of N327-Cter and S324-Cter fragments generated by calpain-1. Top, recombinant tau 1N3R treated with Calpain 1 (2 min); Bottom, tau 1N3R mock digest. Uncropped blots and gels are shown in **Supplementary Figure 8**.

A.

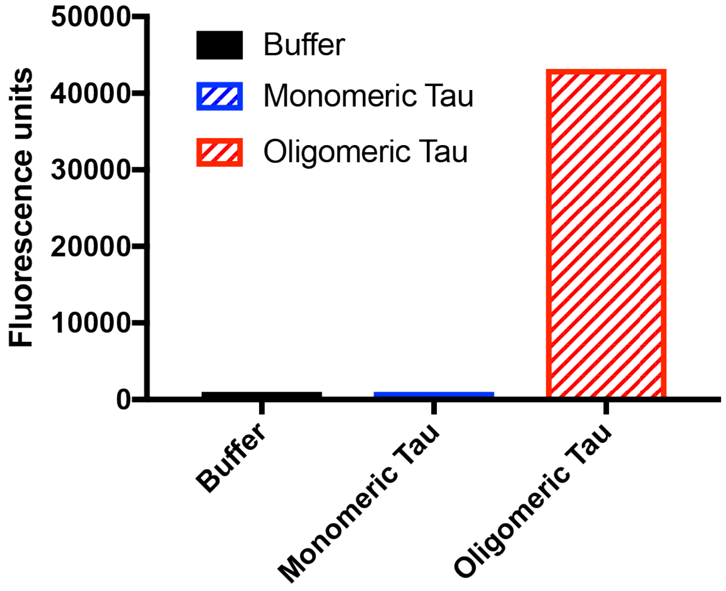

B.

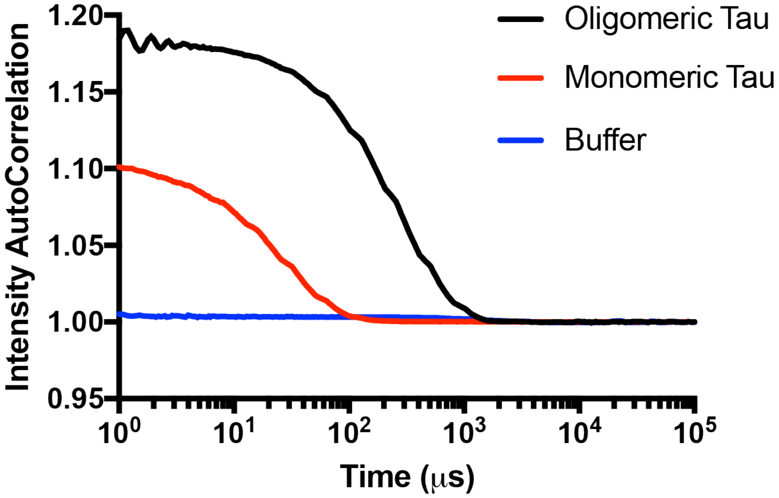

Supplementary Figure 3. Biophysical characterization of oligomeric tau. **a.** Thioflavin T assay of monomeric and oligomeric tau. An elevated fluorescence signal for the oligomeric protein indicates an increase in the beta sheet structure, a signature of the amyloid protein formation of the tau oligomer. **b.** Dynamic Light Scattering autocorrelation spectra of monomeric and oligomeric tau. An elevated scattering signal indicates an increase in the hydrodynamic radius.

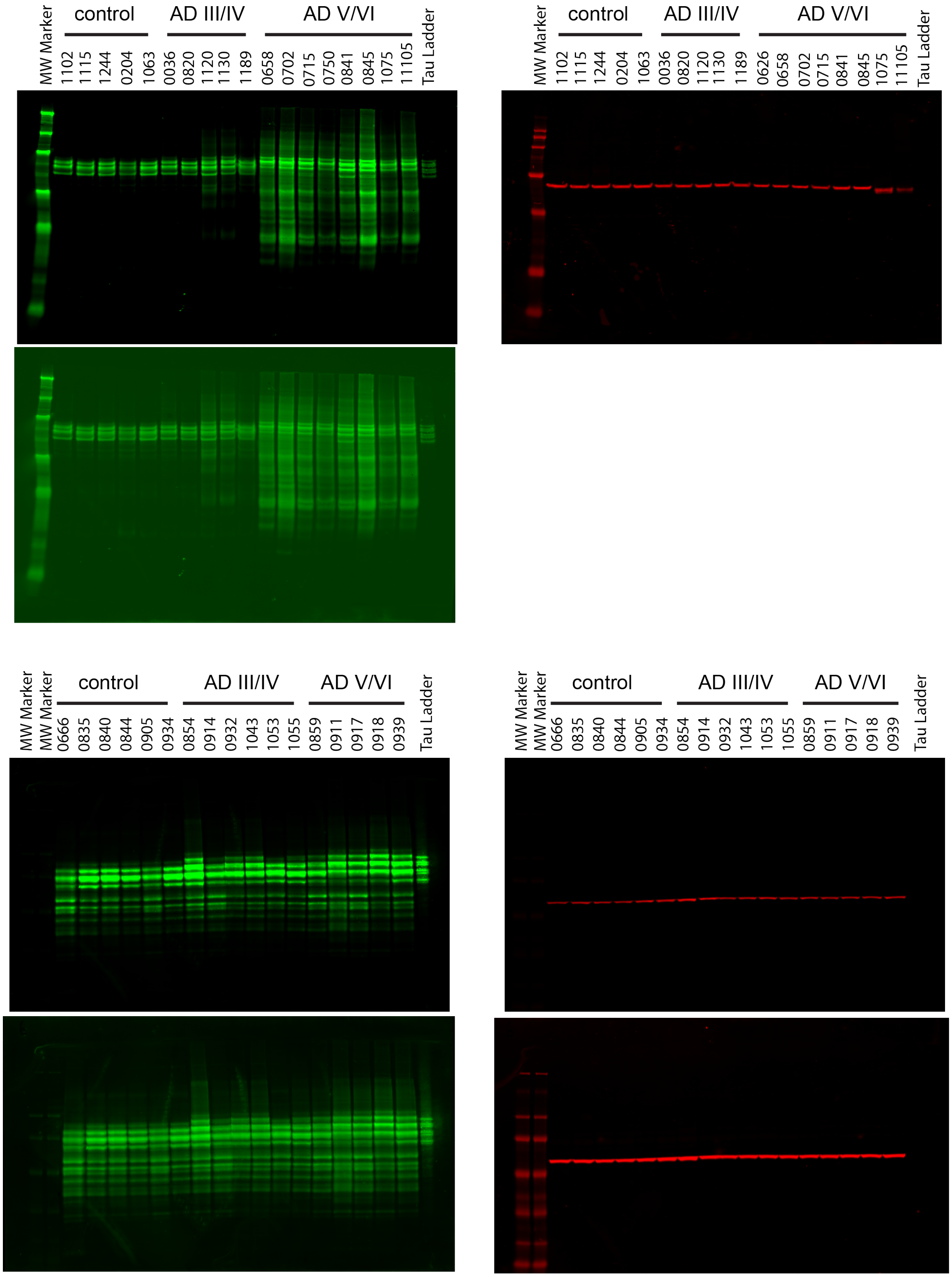

Supplementary Figure 4. Uncropped and additional exposures of immunoblots in Figure 1b (upper) and 1c (lower). Different molecular weight markers were

used in upper (Chameleon Duo, Licor) and lower panels (SeeBlue prestained protein standard, Life Technologies). Sample numbers are labeled for each lane.

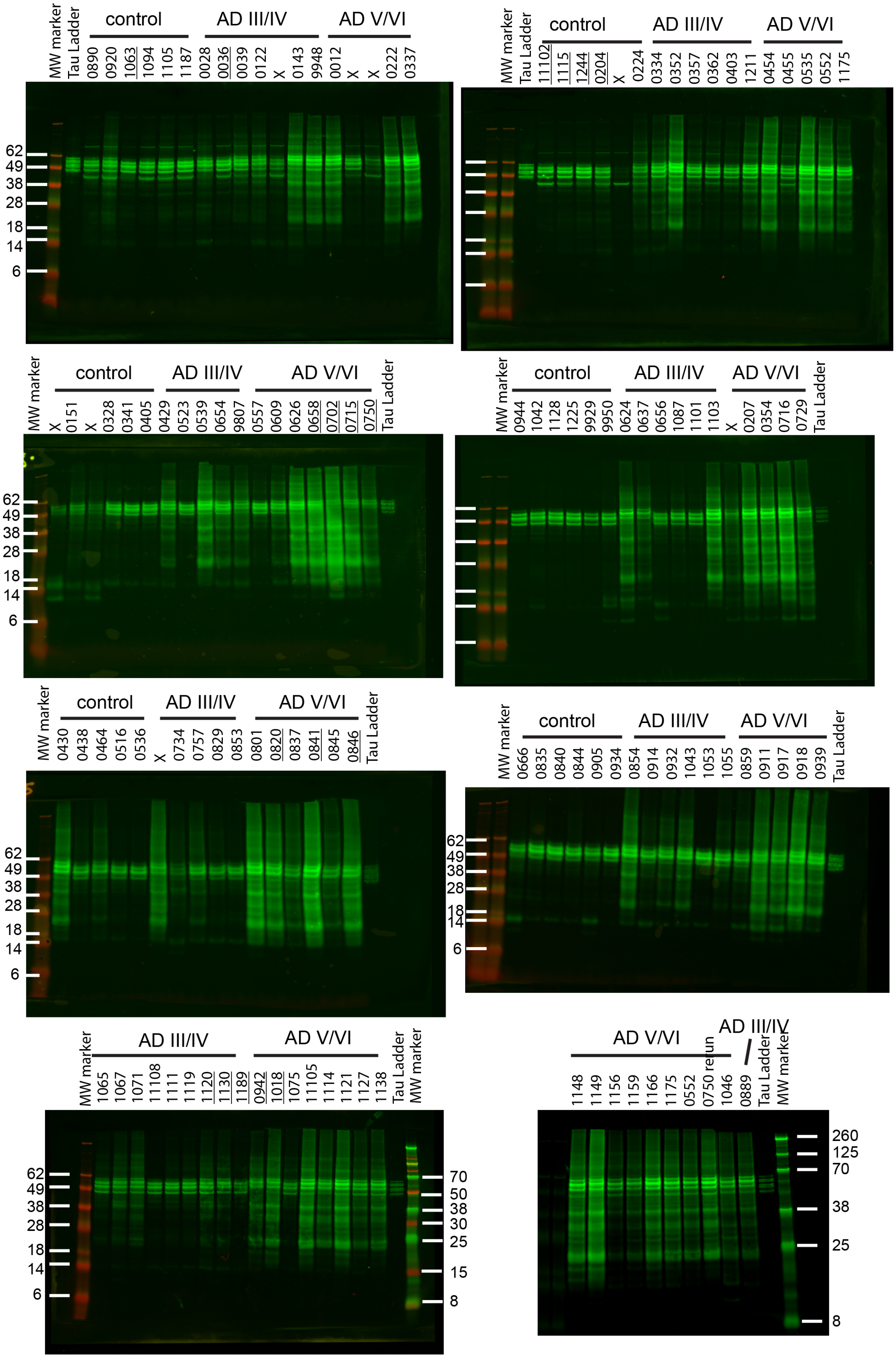
MW marker
Tau Ladder
control
AD III/IV
AD V/VI
0890 0920 1063 1094 1105 1187 0028 0036 0039 0122 X 0143 9948 0012 X X 0222 0337
62 49 38 28 18 14 6
MW marker
Tau Ladder
control
AD III/IV
AD V/VI
11102 1115 1244 0204 X 0224 0334 0352 0357 0362 0403 1211 0454 0455 0535 0552 1175
MW marker
control
AD III/IV
AD V/VI
X 0151 X 0328 0341 0405 0429 0523 0539 0654 9807 0557 0609 0626 0658 0702 0715 0750
Tau Ladder
62 49 38 28 18 14 6
MW marker
control
AD III/IV
AD V/VI
0944 1042 1128 1225 9929 9950 0624 0637 0656 1087 1101 1103 X 0207 0354 0716 0729
Tau Ladder
MW marker
control
AD III/IV
AD V/VI
0430 0438 0464 0516 0536 X 0734 0757 0829 0853 0801 0820 0837 0841 0845 0846
Tau Ladder
62 49 38 28 18 14 6
MW marker
control
AD III/IV
AD V/VI
0666 0835 0840 0844 0905 0934 0854 0914 0932 1043 1053 1055 0859 0911 0917 0918 0939
Tau Ladder
62 49 38 28 18 14 6
MW marker
AD III/IV
AD V/VI
1065 1067 1071 11108 1111 1119 1120 1130 1189 0942 1018 1075 11105 1114 1121 1127 1138
Tau Ladder
MW marker
62 49 38 28 18 14 6
70 50 38 30 25 15 8
AD V/VI
AD III/IV
1148 1149 1156 1159 1166 1175 0552 0750 rerun 1046 0889
Tau Ladder
MW marker
260 125 70 38 25 8

Supplementary Figure 5. Additional uncropped C-ter-1 immunoblots for quantification in Figure 1. Underlines samples are not quantified from these blots but rerun and quantified in immunoblots shown in **Figure 1a**. X-marked lanes were excluded from the analysis due to low overall tau signals.

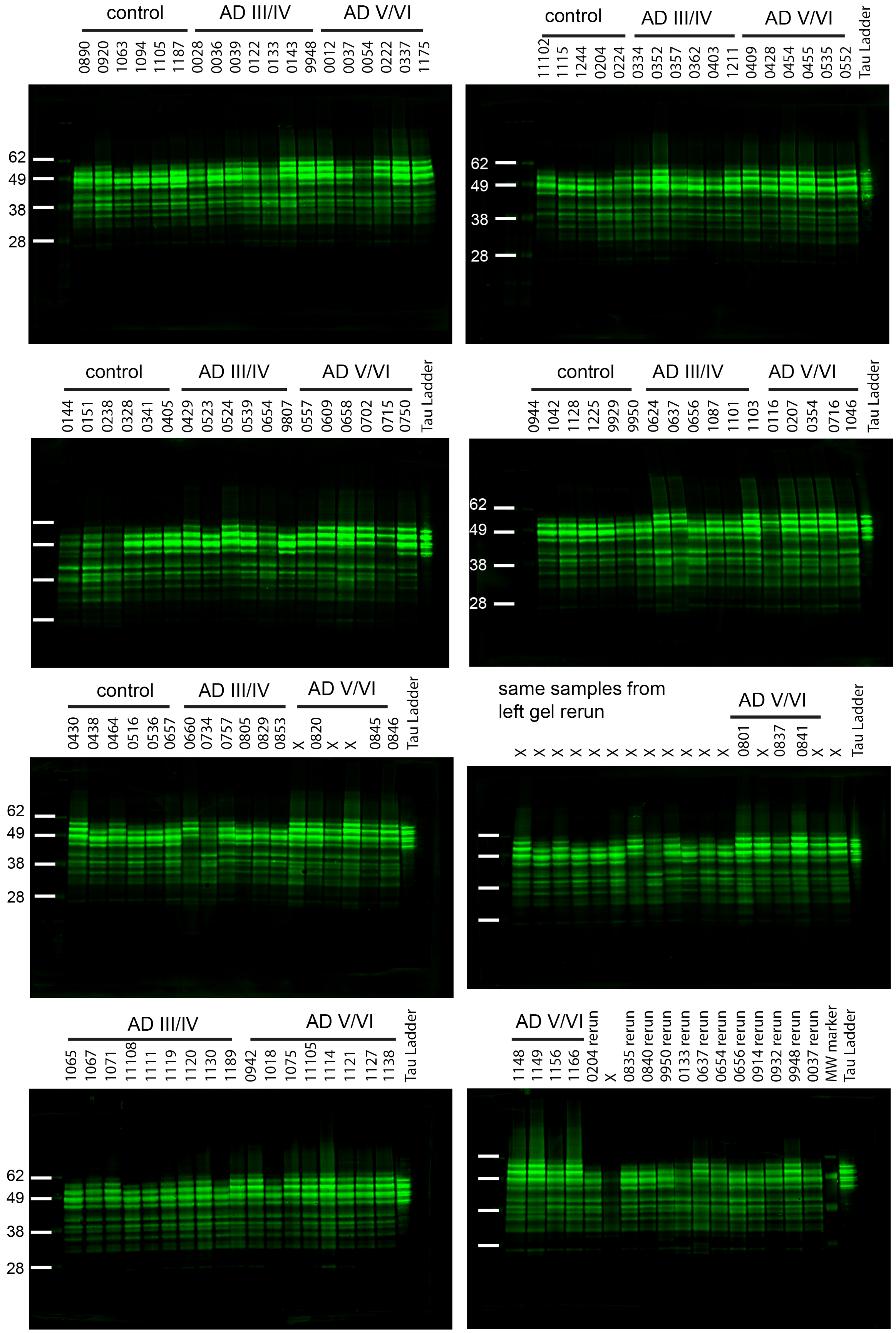
control
AD III/IV
AD V/VI
0890 0920 1063 1094 1105 1187 0028 0036 0039 0122 0133 0143 9948 0012 0037 0054 0222 0337 1175
62
49
38
28
control
AD III/IV
AD V/VI
11102 1115 1244 0204 0224 0334 0352 0357 0362 0403 1211 0409 0428 0454 0455 0535 0552 Tau Ladder
62
49
38
28
control
AD III/IV
AD V/VI
0144 0151 0238 0328 0341 0405 0429 0523 0524 0539 0654 9807 0557 0609 0658 0702 0715 0750 Tau Ladder
control
AD III/IV
AD V/VI
0944 1042 1128 1225 9929 9950 0624 0637 0656 1087 1101 1103 0116 0207 0354 0716 1046 Tau Ladder
62
49
38
28
control
AD III/IV
AD V/VI
0430 0438 0464 0516 0536 0657 0660 0734 0757 0805 0829 0853 X 0820 X X 0845 0846 Tau Ladder
62
49
38
28
same samples from
left gel rerun
AD V/VI
X X X X X X X X X X X X 0801 X 0837 0841 X X Tau Ladder
AD III/IV
AD V/VI
1065 1067 1071 11108 1111 1119 1120 1130 1189 0942 1018 1075 11105 1114 1121 1127 1138 Tau Ladder
62
49
38
28
AD V/VI
1148 1149 1156 1166 0204 rerun X 0835 rerun 0840 rerun 9950 rerun 0133 rerun 0637 rerun 0654 rerun 0656 rerun 0914 rerun 0932 rerun 9948 rerun 0037 rerun MW marker Tau Ladder

Supplementary Figure 6. Additional uncropped Tau13 immunoblots for quantification in Figure 1. X-marked lanes are not used for quantification and the samples were mostly rerun on other blots shown here or in **Figure 1b**, with one sample excluded from the analysis due to low overall tau signals.

Fig. 2a

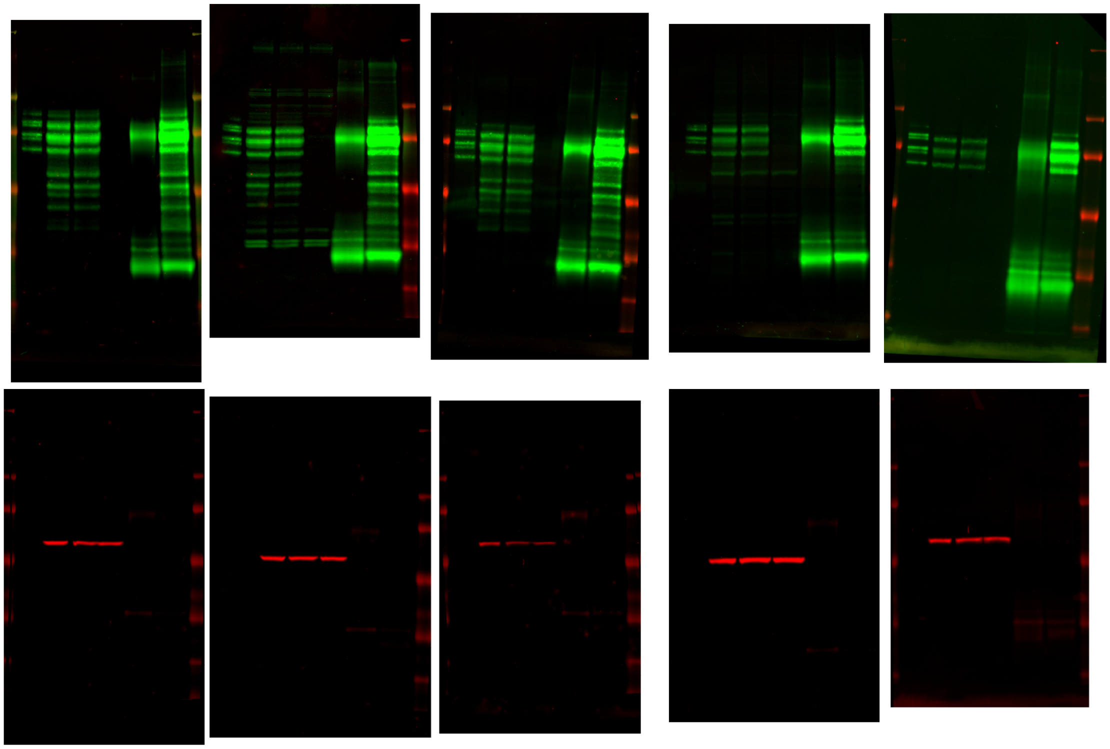

Fig. 2b

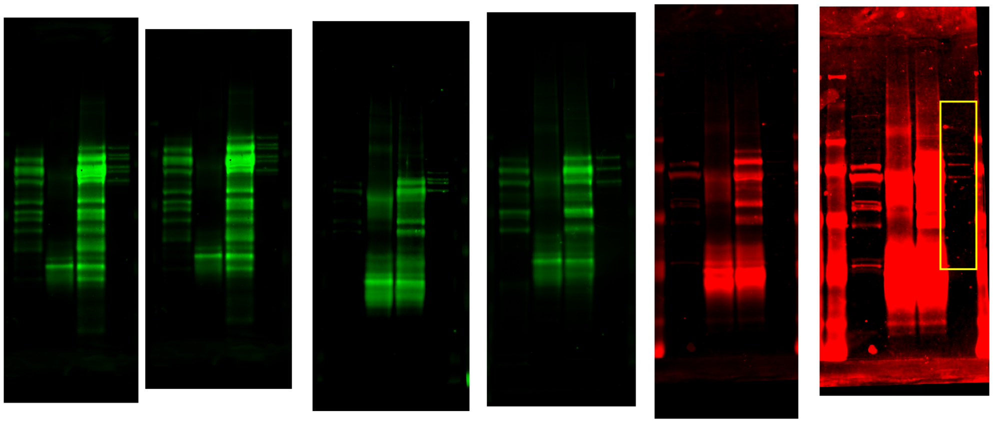

Fig. 2c

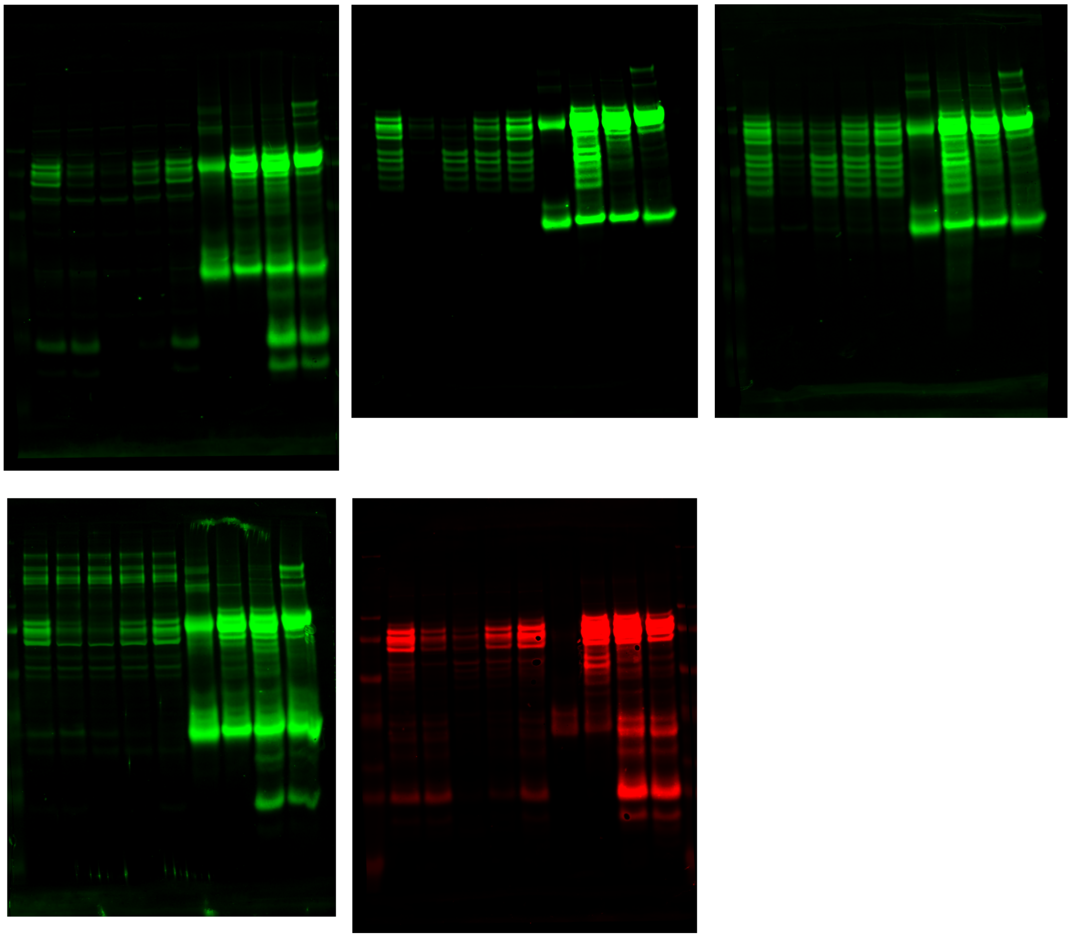

Supplementary Figure 7. Uncropped and additional exposures of immunoblots in Figure 2.

Fig. 3a

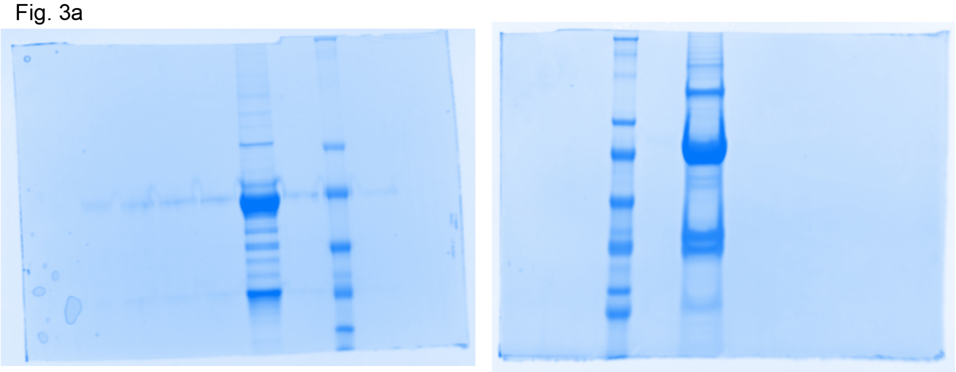

Fig. 3d

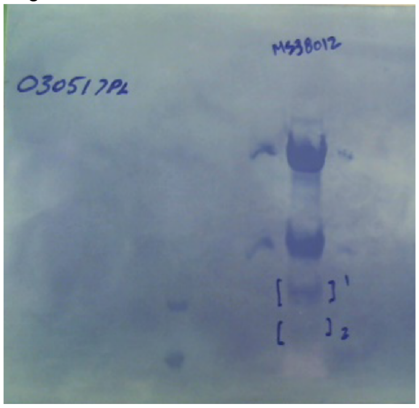

Fig. 4a

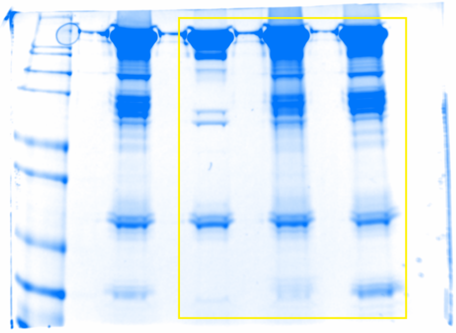

Fig. 5a

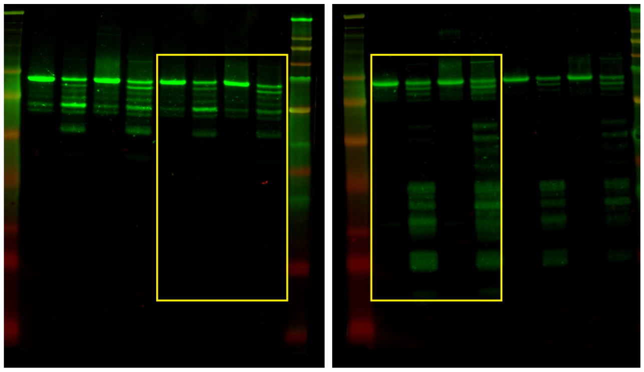

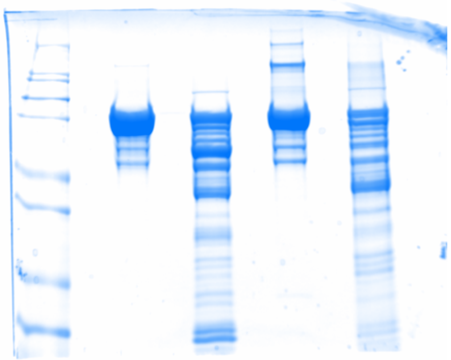

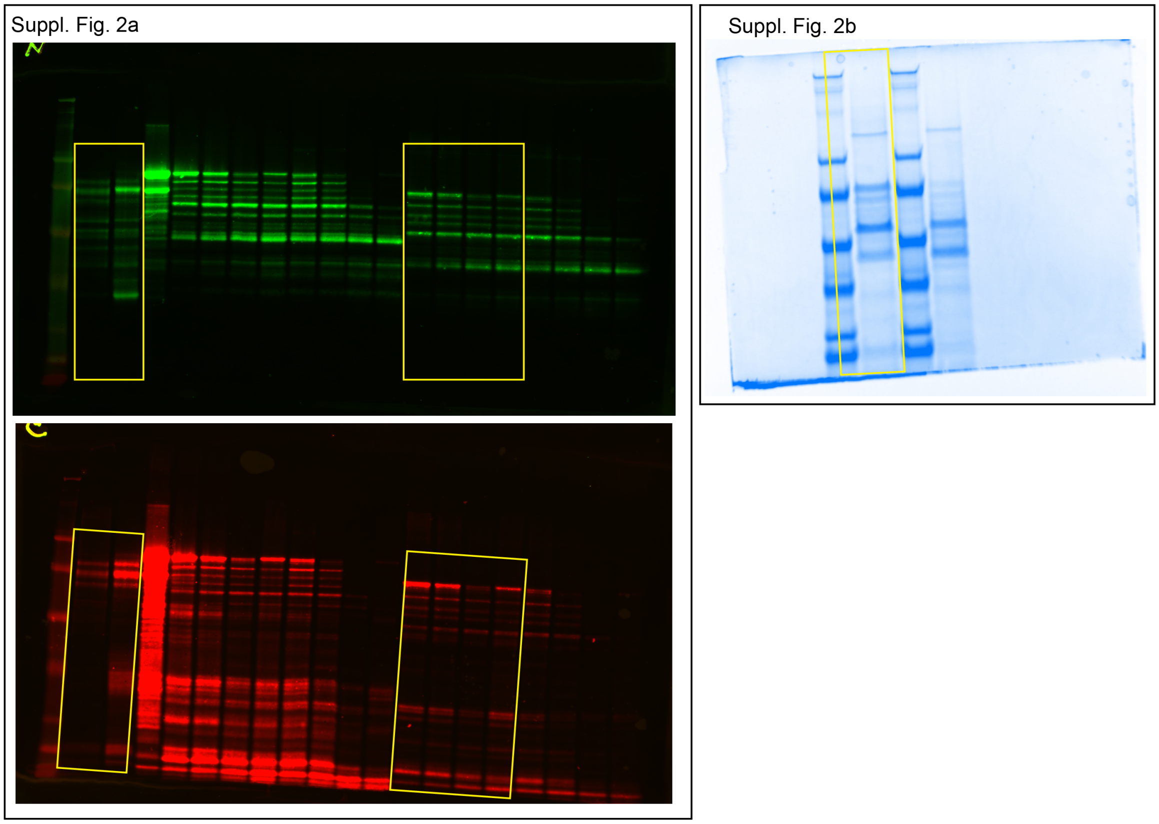

Supplementary Figure 8. Uncropped gels and immunoblots in Figures 3, 4, 5 and Supplementary Figure 2.

Supplementary Table 1.
Tau protease candidates for major fragments identified in this study

Human gene symbol	Degradome	Human description	Mouse gene symbol
**ASPRV1**	A28	aspartic peptidase, retroviral-like 1	Asprv1
**DDI2**	A28	DNA-damage inducible 1 homolog 2 (S. cerevisiae)	Ddi2
**NRIP2**	A28	nuclear receptor interacting protein 2	Nrip2
**NRIP3**	A28	nuclear receptor interacting protein 3	Nrip3
**ADGB**[a]	C02	androglobin	Adgb
**CAPN1**	C02	calpain 1, (mu/I) large subunit	Capn1
**CAPN10**	C02	calpain 10	Capn10
**CAPN12**	C02	calpain 12	Capn12
**CAPN13**	C02	calpain 13	Capn13
**CAPN15**	C02	calpain 15	Capn15
**CAPN2**	C02	calpain 2, (m/II) large subunit	Capn2
**CAPN3**	C02	calpain 3, (p94)	Capn3
**CAPN6**	C02	calpain 6	Capn6
**CAPN8**	C02	calpain 8	Capn8
**CAPN14**	C02	calpain 14	
**ATG4C**	C54	autophagy related 4C, cysteine peptidase	Atg4c
**ATG4D**	C54	autophagy related 4D, cysteine peptidase	Atg4d
**TTC28**	Cx1	tetratricopeptide repeat domain 28	Ttc28
**AMZ2**	M54	archaelysin family metallopeptidase 2	Amz2
**MPND**	M67	MPN domain containing	Mpnd
**FAM111A**	S01	family with sequence similarity 111, member A	Fam111a
**FAM111B**	S01	family with sequence similarity 111, member B	
**MBTPS1**	S08	membrane-bound transcription factor peptidase, site 1	Mbtps1

[a]calpain-like catalytic domain

Supplementary Table 2.
Peptide standards for targeted protein mass spectrometry

Peptide	Parent	Transition	DP volts	CE volts	Standard Level	Curve fit	Mono vs. Oligo calpain digest[a]
**C**GSLGNIHHKPGGGQVEVK	658.670652	998.56292 (3y10)<br>870.467957 (3y9)<br>246.181218 (3y2)<br>218.059388 (3b2)<br>305.091417 (3b3)<br>870.463009 (3z17+2)	79	38.4<br>37.4<br>33.4<br>37.4<br>38.4<br>37.4	High	Quadratic	0.29
SLGNIHHKPGGGQVEVK	586.319948	998.56292 (3y10)<br>870.467957 (3y9)<br>773.415194 (3y8)<br>246.181218 (3y2)<br>778.918238 (3y15+2)<br>201.123368 (3b2)<br>173.128454 (3a2)	73.5	33.5<br>32.5<br>33.5<br>30.5<br>30.5<br>31.5<br>34.5	High	Linear	Mono>Oligo<br>0.00048
NIHHKPGGGQVEVK	500.607429	870.467957 (3y9)<br>773.415194 (3y8)<br>246.181218 (3y2)	67	28.8<br>29.8<br>26.8	HIgh	Linear	Mono>Oligo<br>0.00043
LQTAPVPMPDLK	655.362855	896.491001 (2y8)<br>700.369823 (2y6)<br>472.276575 (2y4)	78.8	27.5<br>36.5<br>37.5	Low	N/A	0.67
LQTAPVP**M**PDLK	663.360313	1084.570708 (2y10)<br>912.485916 (2y8)<br>716.364738 (2y6)	79.4	27.7<br>27.7<br>37.7	Low	N/A	0.28
QTAPVP**M**PDLK	606.818281	912.485916 (2y8)<br>716.364738 (2y6)<br>472.276575 (2y4)	75.1	28.7<br>33.7<br>35.7	Low	N/A	N/A[b]
TAPVPMPDLK	534.791535	896.491001 (2y8)<br>700.369823 (2y6)<br>472.276575 (2y4)	69.6	23.1<br>29.1<br>33.1	Low	N/A	Mono<Oligo<br>0.0038

TAPVP**<u>M</u>**PDLK	542.788992	716.364738 (2y6)<br>472.276575 (2y4)<br>358.686007 (2y6+2)	70.2	29.4<br>33.4<br>29.4	Low	N/A	Mono<Oligo<br>0.0024
APVP**<u>M</u>**PDLK	492.265153	716.364738 (2y6)<br>472.276575 (2y4)<br>358.686007 (2y6+2)	66.4	27.6<br>31.5<br>27.6	Low	N/A	Mono<Oligo<br>0.0029
SGYSSPGSPGTPGSR	697.320762	912.45337 (2y10)<br>815.400606 (2y9)<br>671.347114 (2y7)	81.9	37<br>39<br>34	High	Linear	0.49
YSSPGSPGTPGSR	625.294016	912.45337 (2y10)<br>671.347114 (2y7)<br>456.730323 (2y10+2)	76.5	34.4<br>31.4<br>32.4	Low	N/A	0.64
SSPGSPGTPGSR	543.762351	671.347114 (2y7)<br>416.225208 (2y4)<br>456.730323 (2y10+2)	70.3	32.4<br>23.4<br>27.4	Low	N/A	N/A[b]
GSVQIVYKPVDLSK	511.627607	786.47198 (3y7)<br>658.377017 (3y6)<br>561.324253 (3y5)<br>645.37682 (3y11+2)<br>581.347531 (3y10+2)<br>524.805499 (3y9+2)<br>329.692147 (3y6+2)	67.9	27.4<br>26.4<br>29.4<br>25.4<br>25.4<br>26.4<br>30.4	N/A	N/A	N/A[b]
SVQIVYKPVDLSK	492.620453	786.47198 (3y7)<br>658.377017 (3y6)<br>561.324253 (3y5)<br>645.37682 (3y11+2)<br>581.347531 (3y10+2)<br>524.805499 (3y9+2)<br>329.692147 (3y6+2)	66.4	25.4<br>24.4<br>29.4<br>29.4<br>29.4<br>29.4<br>29.4	High	Linear	N/A[b]
GSVQIVYK	447.258185	650.387188 (2y5)<br>409.244546 (2y3)<br>310.176132 (2y2)	63	20<br>27<br>30	Low	N/A	N/A[b]

SVQIVYK	418.747453	650.387188 (2y5)<br>409.244546 (2y3)<br>310.176132 (2y2)	60.8	18.9<br>24.9<br>23.9	N/A	N/A	N/A[b]
TPSLEDEAAGHVTQAR	561.276185	768.411111 (3y7)<br>574.330736 (3y5)<br>642.302372 (3y12+2)	71.6	33.1<br>33.1<br>29.1	Low	N/A	0.52
IGSLDNITHVPGGGNK	526.945999	628.3413 (3y7)<br>529.272886 (3y6)<br>604.804551(3y12+2)	69	31.3<br>27.3<br>25.3	High	Quadratic	0.17
SLDNITHVPGGGNK	470.244157	628.3413 (3y7)<br>529.272886 (3y6)<br>604.804551 (3y12+2)	64.7	28.2<br>27.2<br>23.2	Low	N/A	N/A[b]

[a]Student's T test of absolute peptide quantities measured, adjusted p value
[b]Below detection limit
